# Disciplining the digital public: Platform mechanisms and the dynamics of emotional polarization

**DOI:** 10.1371/journal.pone.0342143

**Published:** 2026-08-13

**Authors:** Changrong Lu, Dan Qin, Jiali Huang, Bing Pang

**Affiliations:** 1 Nagoya University of Commerce and Business, Nisshin, Aichi, Japan; 2 Shanghai Institutes for International Studies, Shanghai, China; 3 Graduate School of Asia-Pacific Studies, Waseda University, Tokyo, Japan; University of Foggia: Universita degli Studi di Foggia, ITALY

## Abstract

Digital platforms increasingly mediate political communication and public opinion formation, raising urgent questions about how Bigtech firms are associated with behavioral norm formation and affective dynamics. This study develops the concept of platform disciplinary mechanisms to examine how interface design, algorithmic recommendation, and interaction feedback correspond to behavioral standardization, cognitive dependency, and emotionally structured group differentiation across online communities. Using a platform-end user-community framework, we investigate whether technological conditions associated with platform governance correspond to systematic changes in emotional polarization within the platform-mediated information environment. We conduct a time-series analysis linking a daily emotional polarization indicator derived from aggregated news sentiment (GDELT) to technology-category intensity measures extracted from Bigtech research outputs indexed in the Web of Science. Because the polarization indicator is based on news sentiment rather than user traces, it captures emotional polarization in the information environment rather than individual psychological states. Treating 2020 as a theory-informed analytic breakpoint, we find that emotional polarization becomes more persistent after 2020 and is more strongly associated with higher levels of technological simplification and emotion-oriented interaction dynamics. These findings are consistent with the view that major platforms are linked to changes in public-opinion dynamics in platform-mediated environments characterized by affective governance. They also suggest that regulation should pay greater attention to structural mechanisms of behavioral normalization and affective manipulation beyond content moderation alone.

## 1 Introduction

As the world enters the era of Internet 3.0, the Internet has profoundly reshaped the patterns of social interaction and development, bringing diverse forms of information and exchanges into a unified medium [1]. Bigtech firms, exemplified by Apple, Amazon, Microsoft, Google, and Facebook, have become important loci of power in information distribution, user guidance, and social organization, owing to their deep mastery of platforms, data, and algorithms [2]. Since the 2016 U.S. election, these companies have become increasingly involved in political agenda‑setting and emotional mobilization through personalized recommendations, precision targeting, and topic manipulation, becoming pivotal institutional actors shaping democratic public opinion [3,4].

A growing body of research has systematically revealed how Bigtech transforms “users” and “engagement” into commercial assets, influencing attention allocation, behavioral choices, and even value identification through algorithmic optimization and interface design [5–7]. Such practices have not only raised global concerns over user privacy but, due to their escalating political influence, have also sparked widespread worries about platform regulation, civil liberties, and democratic stability [8]. The January 6, 2021, Capitol insurrection underscored this trend: protesters relied heavily on social media platforms to disseminate false information, exacerbating social polarization and political extremism [9,10]. This phenomenon is closely linked to emotionally driven algorithms designed to boost engagement by amplifying emotional reactions, thereby facilitating the spread of extremist views and posing a latent threat to democratic institutions [11,12].

Notably, the dual embedding of technology into political structures and social interactions has not consistently delivered the anticipated benefits of information pluralism and public rationality. Recent research on digital transformation highlights that such transformations often involve fundamental changes to organizational strategies, value chains, and structural mechanisms, beyond incremental technological upgrades. In particular, studies investigating the adoption challenges of Industry 4.0 for sustainable digital transformation argue that digital technologies reshape business models and organizational structures in profound ways, affecting resource flows, collaboration, and socio‑technical interactions [13]. This literature underscores that digital transformation should be viewed not merely as an efficiency tool but as a structural process influencing governance, coordination, and institutional arrangements. Instead, while ostensibly supporting diverse expression, digital platforms have in practice intensified information filtering and echo‑chamber effects [14,15]. This paradox raises a set of critical theoretical questions that have become central to political-social inquiry in the digital age. Digital transformation should not be understood solely as an efficiency-enhancing technical shift. Recent scholarship emphasizes that the digital transition should not be viewed merely as an Industry 4.0 efficiency upgrade, but rather as a fundamental reorganization of the social order that directly impacts social cohesion and human-centric governance, mirroring the concerns raised in the transition toward Society 5.0 [16]. In this broader sense, Bigtech platforms are not merely channels for information delivery but part of a wider structural transformation that links technological change to social coordination, public order, and sustainability-related concerns.

Why do platforms explicitly designed to expand connectivity and exposure to diverse viewpoints increasingly coincide with opinion homogenization, emotional polarization, and social fragmentation? How do Bigtech firms, while actively encouraging participation and interaction, shape the boundaries of public discourse and the emotional conditions under which political communication unfolds? These questions challenge prevailing assumptions about technological openness and require closer examination of the mechanisms through which platforms structure collective attention and affective alignment.

Much of the existing literature approaches these issues from a technology-deterministic perspective, attributing Bigtech’s expanding socio-political influence largely to its control over rapidly advancing technologies, including internet infrastructure, algorithmic systems, smart devices, and the Internet of Things [17]. Within this framework, users are largely conceptualized as passive adaptors whose behavioral patterns and cognitive orientations are shaped by technological architectures that operate beyond individual agency.

At the individual level, a substantial body of research has examined how digital technologies intervene in everyday behavior. Lupton [18] first argued that the widespread adoption of smart devices created the material conditions for “self-tracking,” enabling users to record, quantify, and adjust daily routines through wearable technologies, thereby producing increasingly standardized behavioral trajectories [19]. Similarly, IoT systems connect sensors and interfaces to facilitate real-time monitoring and data collection of user activities; through advanced analytics, platforms construct predictive behavioral models that subtly guide user decision-making [20,21]. While such technology-driven and often unconscious forms of self-monitoring may enhance efficiency and convenience, they can gradually undermine individual autonomy and pose latent risks to trust-based mechanisms within democratic societies [22].

At the collective level, scholars have focused on how algorithmic systems reshape patterns of opinion formation and expression by identifying user preferences, assigning classificatory labels, and mapping social trajectories at scale. Balkin [23] conceptualizes this transformation as the emergence of an “algorithmic society,” in which social valuation and behavioral space are increasingly encoded into platform architectures, enabling the organization of group behavior. By tracing digital footprints, platforms deliver highly customized content streams that narrow informational exposure, encourage homogeneous consumption patterns, and influence political cognition and behavioral choice [24]. Pariser’s [25] notion of the “filter bubble” captures how algorithmic personalization reinforces informational closure and resistance to heterogeneous perspectives, increasing susceptibility to misinformation. Building on this insight, DiFonzo et al. [26] advance the “group echo-chamber” framework, showing how prolonged exposure to consonant viewpoints cultivates exclusionary cognitive biases and intensifies emotional distance between social groups. Recent studies further demonstrate that within algorithmically driven attention economies, emotionally charged and extreme content gains disproportionate visibility, amplifying adversarial sentiment and deepening affective divisions in public discourse [27,28].

Moreover, in the domain of political participation, the diffusion of platform-based technological logic has transformed the Internet into a central arena for political mobilization and collective contestation. Kreiss and McGregor’s [3] theory of “instrumental transformation” highlights how network technologies have shifted from passive channels of communication to active mechanisms that shape political behavior, thereby restructuring both modes of participation and processes of decision-making. In this view, digital infrastructures do not merely facilitate political engagement but help reshape the strategic environment in which political actors operate. Woolley and Howard [29], as well as Vaccari, Chadwick, and O’Loughlin [30], further demonstrate that political bots and precision‑targeted advertising systems have become pervasive in campaigning and agenda‑setting, positioning Bigtech platforms as important architects of political discourse. At the same time, by leveraging their control over users’ data, these firms increasingly operate in domains traditionally associated with state governance. As a result, platforms have begun to challenge the state’s monopoly over social coordination and political order, emerging as influential agents in social governance [31,32].

In summary, scholarship on Bigtech grounded in technological-logic paradigms has systematically indicated how platforms guide individual behavior and cultivate group-level opinion homogeneity through algorithmic governance and large-scale data manipulation. This body of work has been instrumental in clarifying platforms’ influence over behavioral formation, cognitive orientation, and information filtering, thereby offering an important theoretical foundation for understanding how platform power intersects with democratic processes and public discourse. Despite these advances, however, three interrelated limitations remain unresolved.

First, existing studies tend to privilege technological architectures and their external structuring effects while under-theorizing platform agency and strategic intent. In particular, insufficient attention has been paid to how algorithmic design, interaction rules, and content presentation are deliberately institutionalized to encourage users to internalize platform-imposed norms, values, and behavioral expectations [33].

Second, much of the literature remains analytically fragmented across levels of analysis. Research frequently focuses on individual-level preferences, cognitive biases, or emotional expression, yet lacks a systemic account of how interactions among platforms, users, and communities evolve over time. As a result, the dynamic coupling of behavioral dependency, cognitive convergence, and emotional resonance, and its transformation into identity-based dependence and group polarization—has not been fully theorized.

Third, although platforms are widely recognized for connecting like-minded individuals and enabling diverse forms of debate [9], empirical evidence increasingly shows that high-frequency interaction combined with algorithmic curation generates discourse environments characterized by emotional intensity and internal homogeneity [34]. What remains insufficiently explained is the synergistic mechanism linking technological logic, emotional incentives, and community belonging—particularly how users gradually construct dependency-based identities at both cognitive and affective levels, thereby deepening inter-group divisions.

To address these gaps and bridge platform studies with communication research on media effects, this paper integrates five closely related strands of scholarship. Agenda-setting research establishes how news coverage structures issue salience and affective tone in public attention [35]. Framing studies further show how evaluative and emotional cues embedded in news narratives shape interpretation and emotional response [36]. Work on affective contagion and affective publics demonstrates how emotionally charged content diffuses through networks and binds communities together [10,28,37]. Research on echo chambers and filter bubbles explains how algorithmic curation and homophily concentrate emotionally consonant content within communities, intensifying within-group cohesion and between-group polarization [25,37]. Finally, studies of recommendation systems reveal how algorithmic ranking and personalization amplify engagement-salient signals often emotional content, thereby reshaping exposure patterns and accelerating affective dynamics [24,27].

Integrating these strands clarifies the mechanism examined in this study. Affective signals embedded in news content (agenda-setting and framing) enter platform information flows, where algorithmic recommendation systems and interface affordances selectively amplify emotionally salient items. Through social contagion and homophilic sharing, these affective cues can be consolidated into community-level emotional identities, which in turn reinforce emotional polarization. This multi-level synthesis provides the conceptual and empirical justification for linking aggregated news-sentiment indicators to platform-mediated emotional dynamics, thereby grounding the GDELT-based empirical strategy within a coherent communication-theoretic framework.

Methodologically, this study adopts a two-step empirical strategy. First, we construct an NLP-based technology categorization pipeline to extract platform-facing technological signals from Bigtech research outputs indexed in the Web of Science, capturing the evolving intensity of key technology domains relevant to platform design and governance. Second, we implement a time-series analytical framework that links these technology signals to an emotional polarization proxy derived from aggregated news sentiment in the GDELT database.

To assess whether the relationship between technological development and emotional polarization varies across different platform governance logics, we estimate a phase-sensitive dynamic model incorporating a structural breakpoint. This design allows us to test whether shifts in disciplinary regimes are associated with changes in the persistence and transmission of emotional polarization over time.

The remainder of the paper is organized as follows. The Literature Review section reviews the relevant scholarship and develops the theoretical framework, covering technical architecture and behavior shaping, disciplinary practices from panopticon to synopticon, dependency-based identity formation, and emotion-driven dynamics and polarization. The Methodology and Analysis section outlines the methodology and analytical strategy, including the research design, data sources from GDELT and Web of Science, the NLP-based classification pipeline, descriptive statistics, and unit root tests. The Results section presents the empirical findings, including breakpoint identification and VAR estimation. The Discussion section examines the implications of the findings, with particular attention to the interpretation of the observed patterns, the role of non-Western platforms, the methodological transparency of ChatGPT-assisted labeling, and the study’s limitations. The Conclusions, Implications, and Future Works section summarizes the study’s main contributions and discussing their implications for platform governance and future research.

## 2 Literature review

### 2.1 Technical architecture and behavior shaping

#### 2.1.1 Division of front‑end and back‑end technologies in bigtech: User interaction and system support.

Since 2008, Bigtech firms have constructed highly interactive digital ecosystems by integrating a wide array of digital technologies and informational tools [38]. Within these ecosystems, users occupy a dual position as both data generators and algorithmically mediated interaction subjects. Front-end technologies are directly user-facing and primarily responsible for shaping how users perceive, access, and engage with information. Through personalized recommendation interfaces, emotionally responsive interaction designs, and streamlined navigational structures, these technologies substantially reduce the cognitive costs of information search and decision-making, while simultaneously increasing user engagement, convenience, and affective attachment to the platform [39].

In contrast, back‑end technologies operate behind the scenes, often imperceptible to users—providing critical system support. Key components include big‑data analytics, behavioral‑prediction engines, distributed computing frameworks, and deep‑learning models [40]. Their primary functions are to process user data in real time, enhance algorithmic accuracy, and ensure the platform’s operational efficiency and security [41,42]. This clear division of labor between front‑end and back‑end systems underpins simultaneous improvements in user experience and platform performance (see Table 1). Consequently, these integrated platform architectures have evolved into foundational structural conditions that coordinate both micro-level behavioral trajectories and macro-level socio-economic transformations within the digital industry [43].

**Table 1:** Comparative overview of front‑end and back‑end technologies.

Technology Type	Representative Technologies	Functions and Objectives	User Impact and Community Interaction
**Front‑End Technologies**	Personalized recommendation; interface optimization; affective design; mobile applications; virtual assistants	Enhance user experience and interaction efficiency; strengthen dependency and emotional identification	Intensify in‑group interaction frequency and emotional resonance, making users more receptive to unified viewpoints
**Back‑End Technologies**	Big data analytics; behavior prediction; distributed computing; deep learning; database management	Process user data in real time; optimize recommendation algorithms; support platform operations	Increase precision of algorithmic control and content delivery within communities, reinforcing viewpoint homogenization

Source: Compiled by the authors based on Bhatt et al. [44], Miraz [45], and Sharma [46].

#### 2.1.2 Technological simplification: Standardizing user behaviors and patterning community interaction.

Unlike traditional information recipients or independent content creators, users within this ecosystem act simultaneously as data consumers and producers. This dual role means that user behavior is no longer driven solely by personal volition but is continually shaped and optimized within the front‑end technological interaction framework. Front‑end technologies not only determine how end users access and consume information but also, through specific interaction designs, influence how they generate and express content. As back‑end systems’ capacity to analyze and regulate user data improves, behaviors become increasingly predictable and patterned, thus magnifying the effects of front‑end–driven technological simplification.

Technological simplification refers to the extensive deployment of streamlined, intuitive interaction tools, such as “like” buttons and one‑click sharing, that markedly reduce users’ cognitive load in navigating operations, making decisions, and expressing emotions (e.g., emojis, fixed phrases), enabling complex tasks to be executed in fewer steps and enhancing both interaction efficiency and user experience [47,48]. However, despite apparent gains in convenience, simplification embeds users’ actions within standardized, modular, and automated structures, promoting behavioral homogeneity and diminishing expressive diversity [49]. Under this regime, platforms gain greater predictive power over user conduct, while users grow increasingly reliant on platform‑prescribed interaction patterns for content dissemination, curtailing opportunities for exploratory or diverse expression [31]. Consequently, the flow of information and modes of interaction within communities are profoundly altered.

Communities such as interest groups or fan bases on social media serve as key sites of user interaction, and their internal dynamics and affective bonds heavily depend on simplification designs. Under technological simplification, emotional expression within these circles becomes more efficient, formulaic, and convergent. Recent research shows that, compared to complex forms of articulation, users tend to favor simplified emotional tools (e.g., emojis, set phrases, or like/report buttons) to convey sentiment, a mechanism that particularly amplifies negative expressions for their directness and efficiency [50]. When a topic elicits a shared emotion among community members (such as anger or discontent), simplification features further intensify “taking sides” behaviors [51]. Users are more inclined to adopt uniform labels, slogans, or memes to signal their stance rather than engage in nuanced debate. This “take‑sides” dynamic not only accelerates emotionally driven information spread but also transforms expression into a symbolic ritual detached from substantive discussion [52]. At its core, this phenomenon reflects an invisible logic of discipline.

### 2.2 Disciplinary practices: Technological governance from the panopticon to the synopticon

#### 2.2.1 The transition from panopticon to synopticon.

The concept of discipline was first articulated by Foucault to describe the systematic control and shaping of individual behavior within social institutions—such as law, education, and the military—so that conduct aligns with societal norms and ultimately yields widely accepted values and codes of conduct [53,54]. Disciplinary practices have traditionally been understood to operate in two modes: the panopticon and the synopticon, which some scholars regard as successive stages in the evolution of disciplinary regimes [55].

The panopticon model, derived from Bentham’s “Panopticon,” emphasizes how a small number of power holders continuously monitor a larger population, generating a felt sense of surveillance that compels individuals to self‑regulate in accordance with institutional rules [56,57]. In contrast, the synopticon model relies on mutual surveillance among many: individuals are visible not only to the platform but also to one another, experiencing an invisible but pervasive peer‑driven oversight [58]. With the rise of Bigtech, disciplinary mechanisms have become more pervasive and covert within online communities, shifting from panoptic to synoptic modalities.

Online communities formed on the basis of shared interests and extended through social media and other digital channels exhibit hierarchical structures and tightly knit network characteristics [59,60]. In its early stages, Bigtech deployed front‑end algorithmic monitoring and personalized recommendations to shape user behavior unilaterally, manifesting a classic panoptic effect: users internalize platform rules as default norms when algorithms deliver highly targeted content, and they consciously conform to avoid reduced visibility or engagement penalties [61].

However, as user populations swelled and interactions diversified, the unilateral discipline of the panopticon proved less effective in managing complex, emotion‑driven group dynamics [62]. Consequently, Bigtech has increasingly adopted a synoptic approach, leveraging peer‑to‑peer mechanisms to enhance mutual supervision and self‑discipline. By embedding features such as “likes,” comments, and shares into the user interface, platforms enlist users themselves as enforcers of community norms. Kingsbury and Hong [63] observe that public feedback and collective pressure prompt individuals to adjust their behavior to align with the prevailing values and viewpoints of their peer group. Posts that deviate from community expectations often incur negative reactions, downgrading of content, or even exclusion from the circle. Although front‑end advances appear to decentralize oversight, they in fact strengthen emotional cohesion and behavioral conformity among users.

In essence, whether operating as a panopticon or a synopticon, disciplinary practices deploy technology to subtly regulate user behavior, fostering unconscious acceptance of platform rules and the gradual formation of uniform social norms. The deeper impetus underlying these practices—namely, the cultivation of dependency‑based identities and affective control—provides a critical entry point for further analysis of disciplinary mechanisms (see Table 2).

**Table 2:** Comparative analysis of the panopticon and synopticon models.

Dimension	Panopticon Model	Synopticon Model
**Design & Concept**	A centralized watchtower surveils surrounding individuals	A decentralized network of mutual public surveillance
**Power Structure**	Authority is concentrated in a central observer	Power appears dispersed but remains centralized within the platform
**Mode of Surveillance**	Unidirectional, passive monitoring	Multidirectional, active peer-to-peer monitoring
**Manifestation**	Few observe the many	Many observe the few
**Psychological**	Mechanism Individuals self-regulate out of fear of being watched	Groups supervise each other, prompting voluntary behavior adjustment
**Applicable Contexts**	Traditional institutions (prisons, schools, factories) and early Internet environments	Contemporary social media and online communities requiring public participation
**Means of Identity Formation**	Individuals perceive external authority and constrain themselves	Mutual peer pressure and public opinion within the group shape identity
**Emotional Impact**	Extreme emotional responses are rare	Emotional polarization is pronounced
**Fundamental Logic**	Cultivate dependency-based identity through behavior and thought control to achieve social order and management	

Source: Compiled by the authors.

#### 2.2.2 The deep motivation of discipline: Shaping dependency-based identity.

Dependency-based identity refers to an individual’s or group’s sense of belonging that becomes highly bound and homogenized through continuous interaction and cognitive internalization within a particular environment. This identity consists of three core dimensions: behavioral dependence, cognitive dependence, and emotional dependence. These dimensions evolve sequentially and reinforce each other [64,65].

At the behavioral level, users initially adapt to platform rules through standardized interactions such as liking, sharing, and other simplified actions. This process leads to a uniform behavior pattern that diminishes individual differences and strengthens users’ dependence and loyalty toward the platform. Subsequently, based on this behavioral dependence, users progressively adopt the mainstream values of the platform, increasingly rejecting alternative information channels [25]. This cognitive homogenization enhances the platform’s control over the flow of information, allowing it to dominate the direction of social discourse.

Emotional dependence, however, is the core of the disciplinary practice. Under the influence of front-end technologies’ emotional engagement mechanisms, users form a strong sense of community belonging, seeing the platform as an essential medium for emotional expression and social connection [66]. Notably, compared to the traditional Panopticon model, emotional drivers are more pronounced in the Synopticon model. As Bigtech shifts its disciplinary mode from the unidirectional surveillance of the Panopticon to the more group-interactive and peer-supervised Synopticon model, users’ emotional ties and collective emotional consensus are effectively strengthened [58]. Users’ emotional identification with the platform’s mainstream viewpoints makes them more likely to consciously support and propagate the topics defined by the platform, further consolidating the platform’s discursive power in public opinion [67].

Thus, emotional drive has become a critical byproduct of Bigtech’s shift in disciplinary practice. It extends the platform’s control beyond technological tools to emotional and psychological levels, making the regulation not only a matter of behavior but also of internalized emotional alignment.

### 2.3 Emotion-driven dynamics: The double-edged sword of unified public opinion and emotional polarization

The analysis above highlights that the core motivation of Bigtech’s disciplinary practices lies in shaping users’ dependency-based identity, turning them into strong supporters of the platform’s discursive power [68–70]. This unified public opinion is heavily reliant on an emotion-driven logic. Front-end technologies, through emotional feedback mechanisms, enhance users’ emotional satisfaction and sense of community belonging, making emotion-driven interactions the norm within these groups. In this context, users increasingly neglect the diversity of information, and cognitive and emotional homogeneity within these groups grows, leading to irrational group psychology and behavior patterns [71,72].

This emotion-driven interaction logic explains the decline of rational expression and the rise of emotional dissemination in online spaces. Compared to traditional communication models, online platforms increasingly depend on emotional resonance and interaction to spread information. Negative emotions, such as anger, fear, and dissatisfaction, because of their high arousal and attentiveness, are more readily accepted and shared by users, thus exerting a more direct and powerful influence on individual attitudes and behaviors [37]. Negative emotions, such as anger and fear, trigger stronger physiological and psychological reactions and, therefore, gain more widespread attention and higher levels of participation on platforms [10]. Research has shown that the spread of anger, in particular, is rapid and widespread, contributing to what is referred to as the “digital anger” phenomenon, where users, due to the emotional stimulus, are more likely to engage in the propagation of anger [73]. The platform’s dynamic recommendation algorithms further amplify emotional interactions among users by adjusting content push strategies in real-time, thereby increasing the rapid spread of negative emotions on social media [74].

Under the Synopticon model, the spread of negative emotions is influenced by multiple interrelated mechanisms [75,76], such as multi-directional information flow and the “echo chamber” effect. These effects cause emotions to accumulate rapidly within the group, ultimately leading to emotional polarization [77,78]. Furthermore, the spread of anger has been shown to promote homogeneity effects, intensifying emotional division between groups [79]. This mechanism not only strengthens emotional homogenization within groups but also fosters cognitive biases and hostility between opposing groups [80]. Therefore, the preferential spread of negative emotions on social platforms is not merely the result of algorithmic biases but reflects human psychological structures’ inherent sensitivity to threat information, ultimately driving the spread of emotionally charged, polarized, and adversarial content. As the disciplinary model shifts from the Panopticon to the Synopticon, users develop stronger internal cohesion through group interaction and emotional resonance, but this also exacerbates emotional conflict between groups and shrinks the space for rational discussion. This transformation not only reshapes the structure of social public opinion but also poses a challenge to the long-term sustainability of Bigtech’s disciplinary mechanisms.

**Fig 1:**
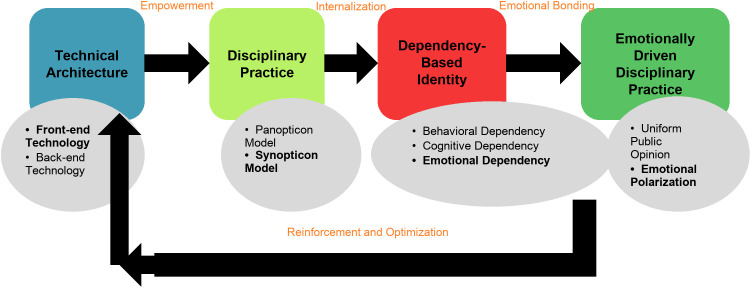
Theoretical framework of Bigtech’s disciplinary mechanisms. Source: Created by the authors.

In conclusion, Bigtech’s use of technological simplification and disciplinary practices has significantly contributed to the standardization of user behavior patterns and reinforced emotion-driven interactions, resulting in the emotional polarization in the platform-mediated public sphere (Fig 1). In sum, the theoretical framework specifies a set of directional and time-varying implications that cannot be settled by conceptual reasoning alone. In particular, the model suggests that (a) platform-facing technologies that lower expressive cost and heighten engagement-salient feedback should be associated with stronger affective divergence, and (b) once platform governance shifts from one-way monitoring to interaction-amplified peer surveillance, affective dynamics may become more self-reinforcing over time. These implications are empirically contestable: their magnitude, timing, and even sign may vary across technological domains and institutional phases. We therefore move to an empirical strategy that tests the model’s observable implications by linking technology-category signals to a time-series measure of emotional polarization, and by examining whether the estimated relationships differ before and after a structural transition in platform disciplining mechanisms.

## 3 Methodology and analysis

This section tests the observable implications of the platform disciplining mechanisms framework in a time-series setting. Rather than restating the research questions developed earlier, we focus on two empirical expectations derived from the theory. First, if platform-facing technological development strengthens disciplining capacity, shifts in technology-category intensity should co-move with changes in emotional polarization in the platform-mediated information environment. Second, if disciplining regimes differ, the technology-polarization relationship should vary across phases, with stronger associations after the hypothesized regime transition.

To evaluate these expectations, we construct a matched daily time-series that links (i) an emotional polarization proxy derived from aggregated news sentiment signals and (ii) platform-facing technology-category intensity measures derived from Bigtech research outputs. We then estimate a dynamic model with a structural breakpoint and report baseline estimates alongside diagnostic checks.

### 3.1 Data collection and processing

Building on the analytical framework established above, this section operationalizes emotional polarization and technology-category intensity in a manner consistent with the paper’s theoretical claims.

The proxy for emotional polarization is derived from the GDELT Global Event Database, which provides large-scale, time-stamped sentiment information for news events worldwide. Specifically, we extract daily sentiment scores associated with reported events and compute the cross-event dispersion of sentiment on each day. The standard deviation of daily sentiment scores is used to capture the degree of affective divergence in the information environment, with higher dispersion indicating greater emotional polarization across contemporaneous news coverage. We adopt the cross-event standard deviation as the dispersion measure for three reasons. First, SD is the canonical second-moment summary of a distribution and aligns directly with the variance-based notion of affective divergence used in prior aggregate sentiment work [81]. Second, GDELT’s sentiment scores are continuous and approximately symmetric around zero, conditions under which SD is informative and not dominated by skewness artifacts that would otherwise motivate Gini- or IQR-based alternatives. Third, the first-differencing operation we apply (yielding dstd) and the additional winsorized and spike-excluded specifications reported in the robustness checks below already absorb the principal robustness concerns that Gini/IQR would address—namely, sensitivity to extreme values. We acknowledge that fully replicating the analysis with rank-based dispersion (Gini, IQR) would provide additional reassurance and identify it as an avenue for future extension. During data preprocessing, observations with abnormal timestamps, duplicated records, or insufficient daily event counts were excluded to ensure the reliability of the dispersion measure. Specifically, we define “insufficient daily event counts” as days with fewer than 500 events, which are excluded to stabilize the dispersion-based sentiment measure and avoid volatility driven by extremely small samples.

After cleaning, the final dataset consists of 2,475 daily observations, covering the period from February 2015 to December 2021. The date-indexed daily series used to construct the empirical variables are provided in S1 Data; the first-difference polarization proxy (dstd) and model-ready inputs are reproducibly generated from these series using S1 Code. This time span captures both the expansion phase of platform-centered political communication and the subsequent period in which emotionally driven interaction dynamics became increasingly salient, thereby providing an appropriate temporal window for assessing the structural impact of platform disciplinary mechanisms on emotional polarization.

Although GDELT captures event-level and media-level sentiment rather than the emotional states of individual social-media users, there are well-established theoretical and empirical pathways that justify its use as an indirect indicator of platform-amplified affective dynamics. Classical agenda-setting and framing research demonstrates that the emotional valence embedded in news coverage shapes issue salience and the interpretive frames available to audiences, thereby influencing subsequent perception and evaluation [35,36]. In the platformized media environment, digital platforms no longer merely coexist with news media but function as algorithmic aggregators and redistributors of news content: headlines, frames, and affective cues originating in the news ecosystem are selectively prioritized, reformatted, and recirculated through personalized feeds and recommendation systems [3,30].

A growing body of experimental and large-scale observational research further shows that emotionally valenced content seeded in news flows readily enters social media streams, where it affects user attention, emotional response, and engagement patterns [10,28]. These findings support a mediated transmission pathway in which affect travels from news production to platform curation and subsequently to user interaction. Taken together, the literature implies a sequential mechanism—news emotion → platform aggregation and recommendation → user exposure → affective contagion and community consolidation, through which media-level affective signals become socially consequential. Under this mediated pathway, systematic changes in aggregated news sentiment (as captured by GDELT) can be interpreted as a co-moving indicator of the affective inputs that platforms ingest, amplify, and circulate. Accordingly, changes in aggregated news affect (as captured by GDELT) are interpreted here as shifts in the affective inputs circulating in the platform-mediated information environment. Importantly, this proxy does not measure individual users’ psychological states; it captures variation in affective tone at the media level that platforms can ingest and amplify through ranking and redistribution.

Technological categorization data were retrieved from the Web of Science platform, limiting the search scope to papers published by five tech giants—Apple, Amazon, Facebook, Google, and Microsoft—between 1975 and 2023. To match the time span of the emotional polarization data, only papers published from 2015 to 2021 were retained, with precise publication dates supplemented through web scraping. To align publication outputs with the daily GDELT timeline, we aggregate category-level publications into daily counts and construct daily technology-intensity series. Because publication events are sparse at the daily level, we apply a *k*-day moving-average smoothing (*k* = 7) to reduce day-to-day zero-inflation noise in the intensity series, rather than imputing publication dates. This procedure yields a complete dataset containing 49,775 paper abstracts. Because WoS-indexed publications are publicly visible research outputs rather than direct records of internal technology deployment, the technology-intensity series should be interpreted as research-output signals of broader technological conditions. Published research may lag internal R&D or deployment decisions by one to three years; therefore, the observed post-2020 associations may partly reflect R&D trajectories initiated earlier and subsequently becoming visible in the publication record. We thus interpret these variables as indicators of evolving platform-facing technological emphasis, rather than as immediate measures of feature deployment.

We further note that prior work using platform-trace emotion data has consistently identified emotional amplification and polarization as platform-level dynamics on social media. For instance, Brady et al. [82] showed that moralized emotional language on Twitter predicts higher diffusion and engagement, indicating how emotional content shapes network propagation dynamics. Bail et al. [83] found that exposure to opposing views on social media platforms such as Twitter or Facebook can paradoxically increase affective polarization rather than reduce it. Rathje et al. [84] demonstrated that content expressing out-group animosity on Facebook and Twitter strongly predicts engagement metrics, suggesting that platform architectures inherently amplify certain emotional expressions. Compared with these proprietary Twitter or Facebook emotion datasets, GDELT’s news-sentiment dispersion proxy operates upstream of within-platform engagement traces: it captures the affective tone of news inputs that platforms ingest and redistribute rather than the user‑level engagement signals themselves. Relative to proprietary trace-level emotion indicators, GDELT offers public availability, longitudinal continuity, and replicability, at the cost of greater distance from individual user affective states. We view this as a deliberate analytical trade-off rather than a substitute for trace-based studies.

Ethics statement. This study used aggregated media-level data from GDELT and bibliographic research records obtained through Web of Science. It did not involve the recruitment of or interaction with human participants, the use of human biological materials, or access to identifiable private personal data. Accordingly, institutional ethics approval and informed consent were not required.

### 3.2 Technology categorization and time-series construction

We identified Bigtech-affiliated publications in Web of Science using a predefined firm list and affiliation-based search strings. Records were then mapped into ten technology categories using a two-stage NLP pipeline that combines unsupervised clustering with active-learning-assisted labeling. Category assignments were subsequently aggregated into a time-aligned technology-intensity series matched to the GDELT daily timeline. This construction is intended to capture temporal variation in the prominence of platform-facing technology domains in Bigtech firms’ research portfolios.

The NLP procedure treats each article abstract as one text unit. After text preprocessing, abstracts were converted into SentenceTransformer-based semantic embeddings. UMAP dimensionality reduction was then applied before clustering. The initial technology categories were generated using MiniBatchKMeans, with the final number of clusters set to 10 (k = 10). This cluster number was selected to balance thematic granularity, category interpretability, class size, and alignment with the theoretical distinction between user-facing and infrastructural platform functions. TF-IDF or class-based TF-IDF keywords were used to interpret the clusters rather than as the primary clustering features.

To refine the initial categories, we implemented an active-learning-assisted labeling procedure. In each round, a small set of low-confidence abstracts, defined by the lowest predicted category probabilities or ambiguous category membership, was selected for review. The active-learning process was conducted over six rounds, with approximately 30 low-confidence abstracts reviewed per round. ChatGPT was used as an auxiliary labeling and category-interpretation tool in the later rounds, following a pre-specified labeling guideline and prompt template. The authors reviewed the category interpretations and used the reviewed labels to improve the classifier. ChatGPT was not treated as an independent human coder; rather, it functioned as a model-assisted review tool for boundary cases and category interpretation.

The final categories were interpreted using representative keywords, sample abstracts, and the active-learning review process. For example, Type0 captures security, privacy-preserving computation, and trust infrastructure; Type3 captures runtime systems and dynamic software infrastructure; Type4 captures social platforms, search, and user activity; and Type5 captures image processing and visual-content labeling. These categories were then mapped onto platform-disciplinary functions as an interpretive bridge between Bigtech research-output signals and the theoretical framework. The full Web of Science retrieval strategy, screening protocol, feature-construction procedure, clustering settings, prompt template, validation notes, and technology-category mapping are provided in S1 Text. The date-indexed daily series used to construct the empirical variables are provided in S1 Data. All code used for data processing, technology categorization, time-series construction, and statistical analysis is provided in S1 Code to ensure reproducibility.

## 4 Results

### 4.1 Descriptive analysis and stationarity test

Based on the keywords generated from clustering (see Table 3) and Rodríguez and Ortún’s [38] front-back technology framework, the 10 technology categories are provisionally interpreted as two major dimensions:

**Table 3:** Ten technology categories: Representative keywords, front-/back-end orientation, and primary links to platform disciplinary mechanisms.

Technology category	Representative keywords (TF-IDF)	Orientation	Primary disciplinary mechanism
Type 0 — Privacy, security & trust infrastructure	security, integrity, malicious, secure inference, MPC, protocol	Front-end	Cognitive dependency
Type 3 — Runtime & dynamic recommendation systems	type system, polymorphic, runtime, dynamic, parameterized	Front-end	Behavioral standardization
Type 4 — Social platform, search & user activity	social, platform, user, activity, search, information-seeking	Front-end	Cognitive dependency
Type 5 — Image processing & visual labeling	image, pixel, labeling, visual, CRF, neighborhood	Front-end	Emotional differentiation
Type 1 — Query optimization & workload infrastructure	big data, workload, optimizer, complex query, decision	Back-end	Behavioral standardization (supporting)
Type 2 — Neural-network modeling & tuning	DNN, activation, hidden layer, neural, parameter	Back-end	Cognitive dependency (supporting)
Type 6 — Access control & policy management	security, user, transaction, control, policy, access	Back-end	Behavioral standardization (supporting)
Type 7 — Code & computational-method engineering	code, pairwise, geometry, experiment, boundary	Back-end	Behavioral standardization (supporting)
Type 8 — Motion & spatiotemporal signal processing	motion, velocity, spatiotemporal, phase, signal	Back-end	Emotional differentiation (supporting)
Type 9 — Ad auctions & attention allocation	auction, slot, allocation, position, revenue, click	Back-end	Emotional differentiation (supporting)

Note. Categories are derived from a SentenceTransformer–UMAP–MiniBatchKMeans (k = 10) pipeline applied to 49,775 Bigtech-affiliated Web of Science abstracts; keywords are top TF-IDF/ class-based TF-IDF terms per cluster (S1 Text). For readability, each category is mapped to a single primary mechanism among behavioral standardization, cognitive dependency, and emotionally structured differentiation; “(supporting)” denotes back-end categories that enable rather than directly enact a mechanism. The full keyword lists, representative abstracts, and the complete category-to-mechanism mapping are provided in S1 Text.

(1) **Front-End Technologies (Type 0, Type 3, Type 4, Type 5)**: These technologies are more directly related to user-facing interaction and content presentation. For instance:

Type 0 is associated with data privacy and encryption-related functions that may enhance user trust;
Type 3 and Type 4 are associated with recommendation- and platform-mediated interaction patterns that shape emotional expression;
Type 5 is associated with image-processing functions that may strengthen the emotional rendering of content.

(2) **Back-End Technologies (Type 1-2, Type 6-9):** These technologies are more closely related to the platform’s underlying computational logic and support functions.

Type 1 is associated with big data optimization
Type 2 with neural network modeling and parameter tuning
Type 6 with user behavior and analysis; and
Type 9 with advertising and allocation algorithms. Collectively, these technologies improve data-processing efficiency and predictive accuracy, and may indirectly shape how emotional content is selected, prioritized, and circulated.

The keyword clusters in Table 3 provide the empirical basis for this interpretive grouping, while the front-end/back-end framework offers the conceptual rationale for distinguishing user-facing functions from system-support functions. The classification is therefore intended as an analytically useful and theory-informed grouping, rather than a claim of exhaustive functional equivalence across all categories. To improve readability, Table 3 provides a compact overview of the ten technology categories, representative keywords, and front-end/back-end orientation; the full mapping to the three disciplinary mechanisms of standardization, dependency, and differentiation is documented in S1 Text.

Based on the above analysis, we obtained the descriptive statistics table for the relevant variables, as shown in Table 4.

**Table 4:** Descriptive statistics of variables.

Variable	Obs	Mean	Std. Dev.	Min	Max
**dstd**	2475	0	.007	−.071	.22
**type0**	2475	.522	.27	.06	1.924
**type3**	2475	.555	.292	.161	2.003
**type4**	2475	.575	.243	.075	1.847
**type5**	2475	.275	.227	0	1.214

Source: Compiled by the authors.

Descriptive statistics show that the mean of the emotional polarization indicator (dstd) is zero, with relatively stable fluctuations (standard deviation = 0.007), but the range of extreme values is large (−0.071 to 0.22), implying localized emotional disturbances. Among front-end technologies, Type 4 (social platforms) has the highest and most stable application intensity (mean = 0.575), while Type 3 (dynamic recommendation) shows significant fluctuations in activity (standard deviation = 0.292, maximum value = 2.003). Type 5 (image processing) experiences periods with zero values (mean = 0.275), which may be related to the cycle of its privacy functions. The extreme value differences in back-end technologies (e.g., Type 0 maximum value = 1.924) reflect phase-specific deployment peaks.

Through the ADF unit root test (see Table 5), it was found that the original emotional polarization indicator (std) is non-stationary (ADF = −1.168, p = 0.687), while its first-order difference series (dstd) passes the stationarity test (ADF = −40.195, p = 0.000). All technical classification variables (type0-type9) are stationary (p < 0.05), meeting the prerequisites for time series modeling.

**Table 5:** ADF unit root test results.

Variable	Test statistic	5% critical value	p-value	Stationary
**Std**	−1.168	−2.860	0.6872	Not stationary
**dstd**	−40.195	−2.860	0.0000	stationary
**Type0**	−4.064	−2.860	0.0011	stationary
**Type1**	−4.373	−2.860	0.0003	stationary
**Type2**	−4.554	−2.860	0.0002	stationary
**Type3**	−4.692	−2.860	0.0001	stationary
**Type4**	−4.646	−2.860	0.0001	stationary
**Type5**	−4.041	−2.860	0.0012	stationary
**Type6**	−4.418	−2.860	0.0003	stationary
**Type7**	−4.420	−2.860	0.0003	stationary
**Type8**	−4.738	−2.860	0.0001	stationary
**Type9**	−4.509	−2.860	0.0002	stationary

Source: Compiled by the authors.

### 4.2 Breakpoint identification and mechanism transition

**Fig 2:**
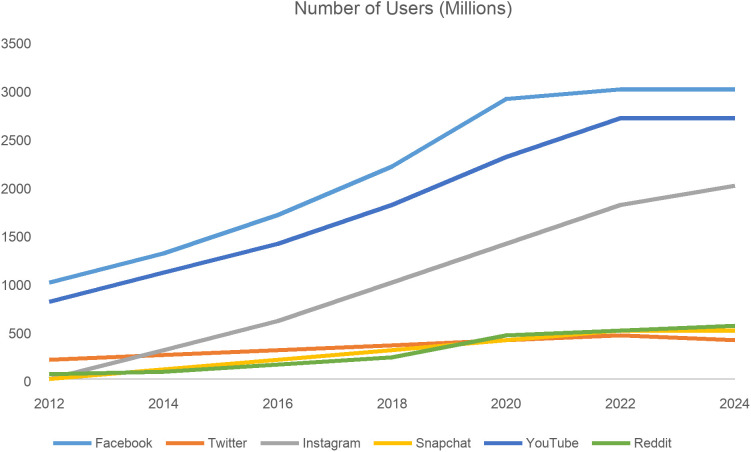
User growth trends of major social platforms (2012-2024). Data Source: DataReportal (accessed 15 Sep, 2025), series compiled and visualized by the authors based on the reported platform user statistics.

Analysis of user growth trends on major social platforms from 2012 to 2024 indicates that 2020 marked a key turning point: prior to this, platforms like Facebook, YouTube, and Reddit relied on a “panopticon” disciplinary mechanism, which monitored user behavior through centralized algorithms and implemented standardized content recommendations, rapidly attracting new users (see Fig 2). After this point, the user growth rate slowed to nearly zero, and platforms shifted to a “synopticon” model, relying on user interactions and group supervision to shape emotional resonance.

From a theoretical perspective, this shift can be attributed to the differences between the two disciplinary mechanisms: the Panopticon relies on one-way algorithmic control to lower the threshold for user participation but struggles to maintain long-term engagement; the Synopticon, on the other hand, requires users to actively adapt to the emotional rules within their circles (e.g., emotional expression and content preferences), making it harder for new users to integrate. Empirical analysis further reveals a significant increase in the persistence of emotional polarization after 2020 (as indicated by the rise in the Dstd L1 coefficient in the subsequent VAR model), suggesting that the Synopticon model amplifies the inertia of emotional transmission through the coupling of technological logic and user interaction. We treat 2020 as an analytic breakpoint motivated by a visible structural change in platform growth dynamics and the broader platform environment. This structural shift is not merely an empirical artifact but aligns with broader observations that digital transitions and algorithmic governance strategies are fundamentally reconfigured when confronting macro-level contexts of profound global uncertainty [85]. Our aim is not to attribute the breakpoint to a single historical event, but to evaluate whether the technology–polarization relationship exhibits phase sensitivity consistent with a regime-dependent disciplining process. We therefore estimate models separately across the pre-2020 and post-2020 phases and interpret differences as regime-contingent associations rather than causal effects.

Substantively, 2020 coincides with at least four platform-relevant developments that jointly motivate its use as an analytic breakpoint: (i) the COVID-19 pandemic shifted public discourse heavily online and increased platform reliance for political information; (ii) major platforms expanded emotional-reaction affordances and short-form video recommendation surfaces during 2019–2020 (e.g., the rapid scaling of TikTok and Reels, the broader rollout of Facebook reaction buttons beyond the like); (iii) the U.S. 2020 election cycle and the January 6, 2021 events brought renewed scrutiny of platform recommendation logics; and (iv) user-growth saturation among major platforms plausibly shifted incentives from acquisition to engagement intensification. We do not claim any single event is dispositive, but the convergence of these structural shifts is what the Chow-type test in Table 6 captures empirically.

**Table 6:** Chow-type structural break test for the 2020 analytical breakpoint.

Breakpoint	Observations used	Pre-break observations	Post-break observations	Tested null hypothesis	Test statistic	p-value	Conclusion
2020-01-01	2,473	1,742	731	Parameter stability across pre- and post-2020 periods	F(6, 2461) = 12.507	< 0.001	Rejected

**Note.** The Chow-type known-break test examines whether the intercept and lagged coefficients in the dynamic model are stable across the pre-2020 and post-2020 periods. The tested model regresses **dstdₜ on dstdₜ**-**₁**, lagged technology-category variables (type0, type3, type4, type5), a post-2020 dummy, and their interaction terms. Robust standard errors are used. Rejection of parameter stability supports the use of phase-specific estimation but should not be interpreted as causal evidence that 2020 itself produced affective polarization. The formal test rejects the null hypothesis of parameter stability*.*

To empirically validate the 2020 breakpoint, we conducted a Chow-type known-break test specifying January 1, 2020, as the analytical breakpoint. The test is based on a dynamic model specification consistent with our VAR framework, regressing dstdₜ on its first lag, the lagged technology-category variables, a post-2020 dummy, and their interaction terms. The robust joint test rejects the null hypothesis of parameter stability across the pre- and post-2020 periods (F(6, 2461) = 12.507, p < 0.001). Substantively, the persistence term (lagged dstd) exhibits the most pronounced shift, rising from 0.149 before 2020 to an implied coefficient of 0.664 after 2020 (detailed interaction coefficients are provided in S1 Appendix). This formal structural break test provides strong empirical support for our phase-specific estimation strategy, though it underscores that 2020 serves as a proxy for a broader structural transition rather than a single causal event.

### 4.3 Empirical analysis based on the VAR model

To ensure comparability across the pre-2020 and post-2020 subsamples, we estimate the same VAR specification in both phases and select the lag order using the same information-criterion procedure (reported in S1 Code). To examine the dynamic impact of technological categorization on emotional polarization, this study constructs a phase-based VAR model with 2020 as the breakpoint. The key results are as follows:

(1) **Persistence of Emotional Polarization:**

The coefficient of the lag term (Dstd L1) increased significantly from 0.149 before 2020 to 0.664 (p < 0.001) after 2020, indicating a stronger self-reinforcing effect of emotional polarization under the shared surveillance model.

(2) **Differentiated Impact of Technological Categorization:**

Frontend Technologies:

Type0 (Data Security) and Type3 (Dynamic Recommendation) showed a significant increase in their positive effects on emotional polarization after 2020, with coefficients rising from 0.000015** and 0.00004 to 0.0021 and 0.0033** respectively.

Type4 (Social Platforms) transitioned from an insignificant impact to a significant positive effect (0.0022**), reflecting the synergistic amplification between algorithmic content push and user interaction.

Type5 (Image Processing):

The coefficient reverses from −0.0008 to a statistically significant negative effect (−0.0035***), which may reflect that certain image-processing and privacy-adjacent design choices reduce the platform’s ability to capture, infer, or amplify users’ emotional signals, thereby dampening affective escalation within platform-mediated interactions.

(3) **Granger Causality Test:**

Before 2020, none of the technological variables passed the significance test (p > 0.05).

After 2020, Type0 to Type5 show significant Granger predictability for emotional polarization (p < 0.05), with Type5 exhibiting the strongest test statistic (Chi2 = 10.672). This pattern suggests that, in the post-breakpoint phase, technology-category signals are not merely contemporaneous correlates but become informative predictors within the modeled dynamics.

### 4.4 Robustness checks for the emotional polarization proxy

To address potential concerns regarding the measurement validity of the emotional polarization proxy derived from news sentiment, we performed a series of sensitivity checks. As reported in Table 7, the core finding that the persistence of emotional polarization increases significantly post-2020 remains robust across multiple proxy specifications.

**Table 7:** Summary of emotional-polarization persistence and selected technology cross-lag associations, pre- versus post-2020.

	Pre-2020	Post-2020	Δ (Post–Pre)
*Panel A. Emotional-polarization persistence (Dstd L1)*			
Baseline (dstd)	0.149***	0.664***	+0.515
Standardized (dstd)	0.149	0.664	+0.515
Winsorized (dstd)	0.350	0.662	+0.312
Spike-excluded (dstd)	0.327	0.635	+0.308
Level (std)	0.998	0.993	−0.005
Winsorized level (std)	0.998	0.993	−0.005
*Panel B. Selected technology cross-lag coefficients (baseline VAR, lag 1)*			
Type0 — security/ trust infrastructure	0.000015**	0.0021**	+0.0021
Type3 — dynamic recommendation	0.000040**	0.0033**	+0.0033
Type4 — social-platform technologies	−0.000137	0.0022**	+0.0024
Type5 — image processing/ privacy	−0.000799	−0.0035***	−0.0027

*Note.* Entries are coefficients from phase-specific dynamic (VAR) models estimated separately for the pre-2020 (n = 1,742) and post-2020 (n = 731) subsamples around the 2020-01-01 analytic breakpoint. Panel A reports the persistence coefficient on the lagged dependent variable (Dstd L1) for the baseline first-difference proxy and five robustness specifications; Δ is the post–pre change. Panel B reports first-lag coefficients of selected technology-category intensities on Dstd in the baseline VAR. * p < 0.05, ** p < 0.01, *** p < 0.001.

The persistence coefficient rises from 0.149 to 0.664 in the baseline first-difference model. This pattern holds when using a standardized dstd measure, a dstd measure winsorized at the 1st and 99th percentiles (rising from 0.350 to 0.662), and a specification excluding the top 1% absolute daily spikes (rising from 0.327 to 0.635). These results confirm that the increased persistence is not an artifact of rescaling, extreme first-difference observations, or isolated spike days. Additionally, level-based specifications (raw and winsorized std) demonstrate high persistence near 1.0 in both phases, confirming the strong memory of the underlying baseline series. Visualizations of these proxy dynamics are provided in the Supporting Information (S1 Appendix).

## 5 Discussion

### 5.1 Findings by time phase

Table 7 summarizes the core phase-specific dynamic results for the two periods separated by the 2020 analytical breakpoint. The first pattern is a clear increase in polarization persistence after 2020. Specifically, the lagged term of polarization (Dstd L1) rises from 0.149 in the pre-2020 phase to 0.664 post-2020 (both statistically significant), indicating that affective divergence becomes substantially more self-reinforcing under the hypothesized synoptic, interaction-amplified regime. Substantively, this increased persistence suggests that under the synoptic regime, emotional contagion is no longer episodic but has formed a self-reinforcing feedback loop, making affective divergence more entrenched within community interactions. More strikingly, translating these coefficients into temporal dynamics implies that the half-life of an emotional shock to the information environment lengthens from approximately 0.4 days to roughly 1.8 days under the post-2020 regime, which is an over fourfold increase in the temporal footprint of any given affective disturbance.

### 5.2 Findings by technology type

The second pattern concerns the differentiated effects of platform-facing technology domains. After 2020, the coefficients for **Type0 (data security-related platform infrastructure)** and **Type3 (dynamic recommendation)** increase markedly and remain positive and significant, consistent with the model’s expectation that scalable data processing and recommendation logic can strengthen engagement-salient exposure patterns. To contextualize these magnitudes substantively: post-2020, a one-standard-deviation increase in Type3 (dynamic recommendation) intensity is associated with a daily change in the dispersion proxy that represents roughly 14% of the overall average daily fluctuation (0.007).

**Type4 (social platform technologies)** shifts from statistically indistinguishable effects before 2020 to a significant positive association afterward, suggesting a stronger coupling between platform interaction design and affective dynamics in the later regime. In contrast, **Type5 (image processing/ privacy-related functions)** becomes significantly negative after 2020. This pattern may reflect that certain image-processing and privacy-adjacent design choices (e.g., reduced granularity of user profiling or targeting precision, and increased friction in content circulation) can dampen affective escalation within platform-mediated interactions.

We interpret this result as suggestive rather than definitive, given that Type5 captures a bundle of visual-content and privacy-adjacent platform capabilities. Overall, these technology-specific findings imply that front-end interaction designs serve as direct amplifiers of affective escalation, whereas reducing the granularity of data capture or affective targeting (as suggested by the Type 5 estimates) represents a potential structural intervention to dampen such polarization.

Third, the Granger predictability tests are consistent with a regime-sensitive interpretation. Before 2020, technology categories do not exhibit systematic predictive power; after 2020, key front-end categories (Type0–Type5) display significant Granger predictability, suggesting that technology signals become more informative for polarization dynamics in the later phase.

These results support a mechanism-consistent reading of the theoretical framework: once disciplining shifts toward synoptic dynamics, emotional polarization shows stronger inertia, and platform-facing technological domains (especially recommendation- and interaction-related categories) are more strongly associated with polarization dynamics. At the same time, the negative association for Type5 highlights the model’s “double-edged” implication: not all technology deepens polarization, and design choices that limit data extraction or targeting may partially offset escalation pressures. These findings have implications for emerging platform-governance frameworks. For example, the EU Digital Services Act requires very large online platforms and search engines to assess systemic risks linked to the design, functioning, and use of their services, including algorithmic systems, recommender systems, advertising systems, and data-related practices [86]. Our results suggest that such regulatory debates may benefit from looking beyond content moderation alone and paying closer attention to structural platform mechanisms, including algorithmic recommendation, interaction-feedback loops, and affective engagement infrastructures. This policy implication should be read cautiously: the present study provides observational and proxy-based evidence, but it highlights why systemic-risk assessment may need to consider the design conditions under which affective polarization becomes more persistent.

### 5.3 Generalizability and broader implications

While our empirical proxy captures generalized media sentiment, the disciplinary mechanisms identified likely operate across diverse digital contexts, though their manifestations may vary. For instance, non-Western platforms like WeChat, which rely heavily on strong-tie social networks, may exhibit different affective contagion dynamics compared to algorithmic-driven short-video platforms like TikTok. The rapid scaling of emotion-reaction affordances and infinite-scroll interfaces on platforms such as TikTok during and after 2018–2020 may form part of the broader platform ecology surrounding the observed post-2020 structural shift. This broader ecology also cautions against treating the Big Five ecosystem as fully representative of global platform governance, since platforms such as WeChat and ByteDance/TikTok may organize interaction, recommendation, and affective feedback through different affordance configurations and institutional logics. Furthermore, the methodological use of AI tools such as ChatGPT for category labeling in our study highlights a broader implication: as algorithms increasingly participate in defining and categorizing knowledge, establishing clear labeling guidelines and human-in-the-loop verification processes becomes essential to prevent the uncritical institutionalization of algorithmic biases.

### 5.4 Limitations

Despite its contributions, this study has limitations that point toward future research directions. First, the empirical analysis focuses on the relationship between platform-facing technological development and an aggregated emotional polarization proxy derived from news sentiment, rather than on individual psychological polarization or platform-internal exposure data. Accordingly, our findings speak to affective dispersion in the platform-mediated information environment (digital public sphere) rather than to users’ internal emotional states; future work could triangulate our proxy with user-trace, survey-based measures, or platform transparency disclosures to better test the proposed micro-to-meso transmission mechanisms. Furthermore, as the GDELT proxy aggregates sentiment signals at a global scale, there is a risk that country-specific effects, particularly those within the U.S. political landscape which is often the focal point of polarization research, may be partially diluted. Due to the lack of access to raw event-level metadata required for localized filtering at this stage, we were unable to perform a formal U.S.-centric robustness check. We acknowledge this limitation and encourage future studies to utilize geographically filtered datasets to evaluate whether these platform disciplinary mechanisms manifest differently across specific national media systems. Second, while this paper emphasizes the effects of platform disciplining mechanisms, subsequent research could explore user heterogeneity in greater depth, examining how different groups accept, negotiate, or resist behavioral and emotional structuring. Third, our phase-based analysis treats 2020 as an analytic breakpoint to evaluate whether the technology-polarization relationship is regime-sensitive; however, this design does not isolate the regime transition from contemporaneous macro shocks, and future work could incorporate alternative breakpoint tests and additional exogenous controls. Additionally, the complex macro-environment inevitably introduces risks of omitted variable bias, and we must acknowledge the potential for reverse causality, where heightened societal polarization may simultaneously drive platforms to develop specific technology categories to manage or capitalize on user engagement. Finally, extending this framework to non-Western or comparative digital contexts would help assess the generalizability of platform disciplining mechanisms and contribute to the development of more inclusive theories of global digital governance.

## 6 Conclusions, implications, and future works

In the context of digital platforms becoming an increasingly central arena for political communication and the reconfiguration of social structures, this study examined how Bigtech platforms are associated with user behavior and emotional polarization through technology-driven mechanisms. By introducing the theoretical framework of platform disciplining mechanisms, this paper developed an integrated analytical model encompassing individual behavior guidance, group identity formation, and the structural patterning of public opinion. Empirically, we observe that emotional polarization becomes more persistent after 2020, and that front-end technology domains related to recommendation and interaction feedback exhibit stronger post-breakpoint associations with polarization dynamics. These patterns are consistent with a regime-sensitive disciplining process operating through platform-mediated affective inputs. Given that the polarization indicator is media-level, our findings speak to affective dynamics in the platform-mediated information environment rather than individual psychological states.

Building on these empirical results, the central finding of this paper is that Bigtech platforms do not merely influence user behavior at the level of technical facilitation. Rather, the coordinated operation of interface design, algorithmic distribution, and interaction feedback is consistent with a broader disciplining process that operates across behavioral, cognitive, and emotional dimensions. At the individual level, this mechanism is associated with gradual dependency on platform logics through standardized interaction paths, simplified decision-making, and the reward of emotionally resonant engagement. At the group level, these mechanisms are consistent with patterns of identity alignment within communities and cognitive segmentation between groups through affiliation signals and affective feedback loops.

This dual process suggests that platforms should not be understood only as neutral intermediaries in information circulation. Instead, they increasingly operate as institutional environments in which social order and identity dynamics are coordinated through interface architecture, recommendation logic, and user feedback. The rise of platform disciplining mechanisms is therefore associated with reconfigurations in the ecology of public opinion in the digital age, including the formation, stabilization, and fragmentation of social consensus. Importantly, the disciplining process identified in this study does not reduce to content filtering or isolated algorithmic control. It reflects a broader governance system in which interface architecture, recommendation logic, and user feedback are jointly associated with patterns of behavioral motivations, cognitive orientations, and emotional structures. As a result, individuals embedded in highly personalized platform environments may increasingly express collective emotions and identity-based alignments, even as their experiences appear individualized at the interface level.

These findings challenge prevailing assumptions about technological neutrality and platform openness that continue to inform much of the policy and public discourse on digital platforms. The power of platforms is not confined to the moderation or amplification of specific content but lies more fundamentally in their systemic alignment with emotional dynamics and identity logics at scale. This systemic alignment helps account for the observed contraction of deliberative spaces, the persistence of emotional polarization, and the deep coupling between platform design and democratic participation. From this perspective, emotional governance emerges as a critical, yet often overlooked, dimension of contemporary platform power.

The implications of these findings extend directly to platform governance. Existing regulatory frameworks predominantly target content moderation, misinformation control, and data privacy protection. While these measures address important risks, they remain insufficient for confronting the deeper behavioral and emotional interventions enabled by platform technologies. The empirical evidence presented here suggests that governance approaches should expand from content-level oversight toward structural accountability for how platforms design interaction mechanisms and emotional feedback systems. In particular, emotional polarization risks should be incorporated into platform responsibility assessments, and platforms should be expected to evaluate the downstream social effects of recommendation and interaction architectures.

In this context, governance strategies may benefit from encouraging the introduction of mechanisms that disrupt excessive behavioral convergence and emotional homogenization, such as diversified exposure pathways or friction in interaction feedback. Moreover, institutional oversight should not rely solely on platform self-regulation. The establishment of neutral and professional supervisory bodies—comprising governmental agencies, academic experts, and technical specialists—could provide independent evaluation of platform disciplining capacities. Such bodies would be positioned to assess how platform technologies co-evolve with behavior, identity construction, and community segmentation, and to integrate these assessments into broader public governance frameworks. By institutionalizing external monitoring of platform disciplining mechanisms, it becomes possible to balance technological innovation and commercial freedom with safeguards against large-scale emotional manipulation and social fragmentation.

These findings point to several extensions that follow directly from our analytical design. Because the proxy operates at the media level, future work could triangulate the GDELT-based indicator with platform-trace data and survey-based measures to test the proposed micro-to-meso transmission pathway more directly. Because it aggregates globally, geographically filtered analyses—particularly U.S.-centric ones—would help recover country-specific dynamics that the global signal may dilute. Beyond the institutional-level account developed here, user-level studies could probe heterogeneity in how different populations accept, negotiate, or resist platform-imposed structuring. Finally, the regime-sensitive pattern we document around 2020 invites tests with alternative breakpoints and extensions to non-Western ecologies such as WeChat or short-video platforms like TikTok, where comparable disciplining mechanisms may operate through different affordance configurations. Rapid scaling of emotion-reaction features and infinite-scroll interfaces on platforms such as TikTok after 2018–2020 may form part of the broader platform ecology surrounding the observed post-2020 structural shift, highlighting that findings for the Big Five ecosystem may not fully generalize to other global platforms, including WeChat and other non-Western platforms. Taken together, these directions would strengthen the empirical foundation for institutional approaches to affective governance and inform comparative theories of digital platform regulation.

Addressing these structural mechanisms of affective governance is therefore a necessary step toward ensuring the broader sustainability of digital transformation, where technological advancement aligns with social resilience and inclusive development rather than undermining them [87].

## Supporting information

S1 Data Daily time-series dataset used to construct the empirical variables. (XLSX)

S1 Text Supplementary methodological documentation for web of science retrieval, screening, clustering, and AI-assisted labeling. (DOCX)

S1 Code Reproducible scripts for data processing and statistical analysis. (ZIP)

S1 Appendix Supplementary results and figures for the breakpoint and proxy-sensitivity analyses. (DOCX)
