# Supplementary material for: Disciplining the digital public: Platform mechanisms and the dynamics of emotional polarization
Source: PLoS One. 2026 Aug 13;21(8):e0342143. doi: 10.1371/journal.pone.0342143 (PMC13472384; doi:10.1371/journal.pone.0342143)
Supplement: Supplementary file 1 [file pone.0342143.s002.docx]

**S1 Text. Supplementary methodological documentation**

*Web of Science retrieval, screening, technology categorization, and AI-assisted active-learning protocol*

This supplementary text documents the data-retrieval and text-classification procedures used to construct the Bigtech technology-category variables. It is intended to improve transparency and reproducibility for the Web of Science search strategy, screening protocol, affiliation-based search strings, feature construction, K-means clustering, active-learning refinement, and technology-category interpretation.

**1. Web of Science retrieval strategy**

The technology corpus was constructed from Web of Science records associated with five major Bigtech firms: Apple, Amazon, Facebook/Meta, Google/Alphabet, and Microsoft. The unit of analysis for the NLP procedure was the article abstract. Titles and abstracts were retained where available; records without usable abstract text were removed before text embedding and clustering. The final categorization dataset contained 49,775 abstracts affiliated with the five firms.

The retrieval strategy combined affiliation-based company identifiers with bibliographic fields. The purpose was to capture scientific and technical research outputs linked to these firms rather than media commentary about the firms. The search was conducted at the document level and then screened for abstract availability, company-affiliation relevance, and duplicate records.

| **Firm** | **Affiliation-based search-string template** |
| --- | --- |
| Apple | AD=("Apple Inc" OR "Apple Computer" OR "Apple") OR OG=("Apple Inc" OR "Apple") |
| Amazon | AD=("Amazon" OR "Amazon.com" OR "Amazon Web Services" OR "AWS") OR OG=("Amazon" OR "Amazon Web Services") |
| Facebook / Meta | AD=("Facebook" OR "Meta Platforms" OR "Meta") OR OG=("Facebook" OR "Meta Platforms" OR "Meta") |
| Google / Alphabet | AD=("Google" OR "Alphabet Inc" OR "Google Research") OR OG=("Google" OR "Alphabet") |
| Microsoft | AD=("Microsoft" OR "Microsoft Research") OR OG=("Microsoft" OR "Microsoft Research") |

*Note: The final query combined the firm-specific affiliation terms above with Web of Science bibliographic filters and abstract-availability checks. The exact field names may vary slightly depending on the Web of Science interface export format. The key principle is that the corpus is affiliation-based rather than topic-keyword-only.*

**2. Screening protocol**

| **Step** | **Screening action** | **Purpose** |
| --- | --- | --- |
| 1 | Retain records with usable titles and abstracts | Ensure that each document can be represented as a text unit for NLP. |
| 2 | Remove duplicate bibliographic records | Avoid over-weighting the same paper across exports. |
| 3 | Screen records by firm affiliation fields | Ensure that documents are linked to the five target firms. |
| 4 | Remove empty, corrupted, or non-informative abstract fields | Reduce noise before embedding and clustering. |
| 5 | Retain abstract as the main text unit | Standardize the unit of analysis for clustering and active learning. |

**3. Text preprocessing**

The NLP pipeline treated each abstract as one text unit. The preprocessing procedure standardized text before embedding and keyword extraction. The preprocessing steps included lowercasing, removal of numbers and non-letter symbols, stop-word removal, and lemmatization. The cleaned abstracts were then used for embedding construction and cluster interpretation.

| **Preprocessing step** | **Description** |
| --- | --- |
| Lowercasing | Converted abstracts to lowercase to reduce case-sensitive variation. |
| Noise removal | Removed digits, punctuation artifacts, and non-letter symbols where appropriate. |
| Stop-word removal | Removed common English stop words and project-specific non-informative words. |
| Lemmatization | Applied lexical normalization to reduce inflectional variation. |
| Abstract-level unit | Kept the abstract as the main document-level text unit. |

**4. Feature-vector construction**

The clustering stage used embedding-based feature vectors. The notebooks used SentenceTransformer models, including paraphrase-multilingual-MiniLM-L12-v2 and bert-base-nli-mean-tokens in different stages of experimentation. The final pipeline used abstract-level embeddings to represent semantic similarity among documents. Embeddings were used for clustering; TF-IDF or class-based TF-IDF keywords were used for cluster interpretation rather than as the primary clustering feature.

To reduce dimensionality before clustering, UMAP was used with cosine distance. The main UMAP settings were n_neighbors = 25, n_components = 10, min_dist = 0.00, and metric = cosine. A fixed random seed was used where possible to improve reproducibility.

| **Component** | **Specification** |
| --- | --- |
| Text unit | Article abstract |
| Semantic feature | SentenceTransformer abstract-level embedding |
| Dimensionality reduction | UMAP |
| UMAP parameters | n_neighbors = 25; n_components = 10; min_dist = 0.00; metric = cosine |
| Keyword interpretation | TF-IDF / class-based TF-IDF top keywords by cluster |

**5. Initial clustering**

The initial technology categories were generated using K-means-style clustering. The analysis used MiniBatchKMeans to improve computational feasibility for the abstract corpus. The final number of clusters was set to k = 10. This value was selected to balance thematic granularity, interpretability, class size, and alignment with the downstream theoretical distinction between user-facing and infrastructural technology functions.

The code explored candidate cluster numbers and used within-cluster sum of squares / elbow-style diagnostics as well as interpretability checks. The MiniBatchKMeans settings included k-means++ initialization, n_init = 512, and batch_size = 1024. Silhouette diagnostics were used as a model-based uncertainty signal to identify low-confidence or boundary cases for later review, rather than as the sole basis for choosing the final number of clusters.

| **Parameter** | **Value / description** |
| --- | --- |
| Clustering algorithm | MiniBatchKMeans |
| Number of final clusters | k = 10 |
| Initialization | k-means++ |
| n_init | 512 |
| batch_size | 1024 |
| Diagnostics | WCSS / elbow diagnostics; silhouette coefficients; interpretability review |

**6. Keyword extraction and category interpretation**

After initial clustering, representative keywords were extracted for each cluster using TF-IDF or class-based TF-IDF. The keyword lists were used to interpret each cluster and to support the active-learning prompt. Cluster labels were therefore not derived from single keywords alone; rather, they were based on the combined evidence from representative terms, sample abstracts, and iterative review of uncertain cases.

**7. AI-assisted active-learning procedure**

The initial K-means categories were refined through an active-learning procedure. Labeled and unlabeled abstracts were combined in an iterative logistic-regression framework. In each round, the lowest-confidence abstracts were selected for review. The project notes indicate a six-round process in which approximately 30 low-confidence abstracts were reviewed per round. The second round was manually checked.

The purpose of ChatGPT in this workflow was to improve the efficiency and consistency of low-confidence sample review. ChatGPT was not treated as an independent human coder. Therefore, the manuscript does not report traditional human inter-rater reliability for the AI-assisted labels. Instead, the active-learning procedure is reported as an uncertainty-sampling and construct-validation step, with the final category labels reviewed against keywords, abstracts, and theoretical mapping.

| **Active-learning element** | **Implementation** |
| --- | --- |
| Initial labels | Generated from the initial 10-cluster K-means solution and keyword review |
| Classifier | Logistic regression within an active-learning framework |
| Uncertainty criterion | Lowest model confidence / ambiguous category membership |
| Samples per round | Approximately 30 lowest-confidence abstracts |
| Number of rounds | Six rounds |
| Human / AI review | Manual checking in the early round; ChatGPT-assisted review in later rounds. |
| Role of ChatGPT | Auxiliary labeling and category-interpretation support, not an independent human coder |

**8. Prompt template for AI-assisted review**

The following prompt template was used to support low-confidence abstract review. It was designed to force the model to rely on cluster keywords and abstract content rather than produce free-form labels.

```
You are assisting with technology-category labeling for Web of Science abstracts from major Bigtech firms. The categories are defined by cluster keywords and example abstracts. Your task is to assign the abstract to one of the existing categories, not to invent a new category.

Input:
1. Abstract text: [INSERT ABSTRACT]
2. Candidate category keyword lists:
   Type 0: [INSERT TOP KEYWORDS]
   Type 1: [INSERT TOP KEYWORDS]
   …
   Type 9: [INSERT TOP KEYWORDS]

Instructions:
- Read the abstract carefully.
- Compare its technical focus with the category keyword lists.
- Choose the best-fitting Type from 0 to 9.
- Provide a short justification based on technical content.
- If the abstract is ambiguous, identify the two closest categories and explain the ambiguity.

Output format:
Assigned Type: [0-9]
Confidence: [High / Medium / Low]
Reason: [One or two sentences]
Alternative Type if ambiguous: [0-9 or None]
```

**9. Mapping technology categories to disciplinary functions**

The final 10 categories were interpreted as technology domains and then mapped onto theoretical functions in the platform-disciplinary framework. This mapping is interpretive and is based on the top keywords generated from clustering, representative abstracts, and active-learning review. The distinction between front-end and back-end functions is used as an analytical heuristic: front-end categories are more directly related to user-facing interaction, visibility, content presentation, and attention allocation, whereas back-end categories are more closely related to computational infrastructure, optimization, security, governance capacity, and system support. Some categories are mixed because they can support both user-facing applications and infrastructural platform operations.

| **Type** | **Indicative category label** | **Representative technical content** | **Disciplinary-function mapping** |
| --- | --- | --- | --- |
| Type 0 | Security, privacy-preserving computation, and trust infrastructure | Security, integrity, malicious detection, plaintext/ciphertext, secure inference, MPC, protocol, component, hardware | Mixed: trust-related platform governance and security infrastructure. Although technically back-end, these tools shape user trust, data protection, and the conditions under which platform interaction becomes acceptable and scalable. |
| Type 1 | Query optimization and workload infrastructure | Big data, workload, optimizer, complex query, decision systems, production optimization | Back-end infrastructure / computational optimization. These technologies support large-scale data processing and platform operational efficiency. |
| Type 2 | Neural-network modeling and parameter optimization | DNN, activation, hidden layer, neural models, signal-to-noise, variables, standards, parameters, environment | Back-end algorithmic capacity. These technologies support model training, prediction, and optimization capacities that can indirectly shape content ranking and platform intelligence. |
| Type 3 | Runtime systems, dynamic programming, and software expressiveness | Type systems, polymorphic/generic instantiation, runtime, dynamic systems, parameterized and expressive programming structures | Mixed: software infrastructure with potential interaction effects. These technologies support dynamic system design and runtime adaptability, which can indirectly enable flexible recommendation, personalization, or interaction architectures. |
| Type 4 | Social platforms, search, and user activity | Social, platform, user, activity, online behavior, search, query, information seeking, choice, seeking | Front-end / user-facing interaction and visibility mechanisms. These technologies are most directly related to platform interaction, search behavior, information access, and user-facing visibility structures. |
| Type 5 | Image processing and visual-content labeling | Image, pixel, labeling, component, visual processing, CRF, conditional minimization, dependency, neighborhood labeling | Front-end content processing / representational filtering. These technologies shape how visual information is recognized, labeled, filtered, and presented in platform-mediated environments. |
| Type 6 | Access control, transaction security, and policy management | Security, user, transaction, control, policy, manager, option, access, balance | Back-end governance / security-control infrastructure. These technologies support platform rule enforcement, secure access, transaction management, and governance capacity. |
| Type 7 | Code modification, computational geometry, and experimental construction | Modifying, code, pairwise checking, geometry, experiment, exponential, boundary, construction | Back-end software systems / computational-method infrastructure. These technologies support system development, code-level optimization, and computational experimentation. |
| Type 8 | Motion, signal, and spatiotemporal processing | Motion estimation, velocity, image signal, spatiotemporal variation, phase, signal processing | Mixed: sensing, signal-processing, and content-analysis infrastructure. These technologies can support back-end analytic capacities while also enabling user-facing applications involving visual, spatial, or temporal content. |
| Type 9 | Advertising auctions, allocation, and monetized attention systems | Auction, slot allocation, distribution, position, revenue, click, advertisement, simulation | Front-end monetization / attention-allocation mechanisms supported by back-end optimization. These technologies are closely related to ad placement, ranking, click optimization, and the allocation of user attention. |

The front-end/back-end distinction should not be interpreted as a rigid classification. Many platform technologies operate across both layers: for example, security and privacy technologies are implemented through back-end infrastructure but affect user trust and participation; neural-network and runtime systems are infrastructural but can enable recommendation and personalization; visual-content processing is user-facing in its effects but depends on back-end classification pipelines. The mapping is therefore used as an interpretive bridge between technology-category indicators and the theoretical concept of platform disciplinary mechanisms, rather than as a claim that each category has only one fixed functional role.

In the empirical analysis, Type 0, Type 3, Type 4, and Type 5 are emphasized because they capture platform-facing domains that connect trust infrastructure, dynamic software systems, search/social activity, and visual-content processing. These categories are not all directly user-facing; rather, they represent different layers through which platform technologies may shape interaction, visibility, content filtering, and affective dynamics. Other categories are retained in the broader classification scheme and reported for transparency, but they are interpreted primarily as infrastructural or governance-support functions. This distinction helps clarify how Bigtech research-output categories are linked to the theoretical mechanisms of behavioral standardization, algorithmic visibility, content filtering, and affective interaction in platform-mediated environments.

**10. Validation, transparency, and limitations**

The classification process combines unsupervised clustering, keyword interpretation, uncertainty sampling, and AI-assisted review. This design improves transparency relative to purely manual labeling while preserving interpretive oversight. Cluster validity was assessed through WCSS / elbow diagnostics, silhouette-based uncertainty review, category interpretability, and consistency with representative abstracts. Because the AI-assisted active-learning procedure was not designed as a multi-human-coder content analysis, traditional inter-rater reliability statistics are not reported. The revised manuscript therefore describes the process as model-assisted category construction rather than conventional human-coded content analysis.
