# Supplementary material for: Disciplining the digital public: Platform mechanisms and the dynamics of emotional polarization
Source: PLoS One. 2026 Aug 13;21(8):e0342143. doi: 10.1371/journal.pone.0342143 (PMC13472384; doi:10.1371/journal.pone.0342143)
Supplement: Supplementary file 2 [file pone.0342143.s004.docx]

**Table appendix 1. Pre- and post-2020 implied coefficients from the known-break model**

| **Variable** | **Pre-2020 coefficient** | | **Post-2020 interaction** | **Post-2020 implied coefficient** | **Interpretation** |
| --- | --- | --- | --- | --- | --- |
| (L.dstd) | | 0.149 | 0.514 | 0.664 | Affective divergence became more persistent after 2020 |
| (L.type0) | | 0.000 | 0.002 | 0.002 | Heterogeneous phase-specific change |
| (L.type3) | | 0.000 | 0.003 | 0.003 | Weak evidence of stronger post-2020 association |
| (L.type4) | | -0.000 | 0.002 | 0.002 | Heterogeneous phase-specific change |
| (L.type5) | | -0.001 | -0.003 | -0.003 | Negative post-2020 shift |

***Note： Post-2020 implied coefficients are calculated as the sum of the baseline pre-2020 coefficient and the corresponding post-2020 interaction term. These coefficient-level results should be interpreted together with the joint Chow-type test. The strongest and most robust shift is observed in the persistence term*** $L.dstd$***, while technology-category coefficients show smaller and heterogeneous changes. Robust coefficient estimates show that*** $L.dstd\times post$***is positive and statistically significant, while*** $L.type5\times post$***is negative and statistically significant.***

**Appendix figure 1. Proxy-Sensitivity Checks for Affective Polarization**

***Note： The figure reports three alternative specifications of the media-level affective-dispersion proxy.*** $dstd$***is the baseline first-difference measure,*** $dstd_{w}$***is the winsorized first-difference measure, and*** $dstd_{z}$***is the standardized first-difference measure. The red dashed line indicates 1 January 2020, the analytical breakpoint used in the phase-specific models.***

**Appendix figure 2. Pre- and Post-2020 Persistence Across Proxy Specifications**

***Note：The figure shows that, across all first-difference-based proxy specifications, post-2020 persistence is higher than pre-2020 persistence. The level-based specifications remain highly persistent in both periods.***
