# Supplementary material for: Disciplining the digital public: Platform mechanisms and the dynamics of emotional polarization
Source: PLoS One. 2026 Aug 13;21(8):e0342143. doi: 10.1371/journal.pone.0342143 (PMC13472384; doi:10.1371/journal.pone.0342143)
Supplement: Supplementary file 3 [file pone.0342143.s006.docx]

**Response to the Editor and Reviewers**

*Manuscript ID: PONE-D-26-02998*

Dear Dr. Cioffi and Reviewers,

Thank you very much for the opportunity to revise our manuscript, "Disciplining the Digital Public: Platform Mechanisms and the Dynamics of Emotional Polarization" (PONE-D-26-02998). We would like to express our deepest gratitude to you and the three anonymous reviewers for the highly constructive criticisms and insightful comments. We have carefully studied all the comments and addressed them through targeted revisions, additional analyses, expanded methodological documentation, and clearer discussion of limitations. We believe that these revisions have substantially improved the clarity, rigor, and theoretical depth of our paper.

First of all, we have carefully reviewed all five Journal Requirements and revised the manuscript and supporting materials accordingly to comply with PLOS ONE's style, file-naming, code-sharing, and submission standards. In specific, regarding Requirement 3, the amended Funding Statement (declaring all sources of support and explicitly noting that no additional external funding was received) is included in our cover letter as requested. Regarding Requirement 5, a complete legend for each Supporting Information file (S1 Text, S1 Appendix, S1 Data, and S1 Code) has been added at the end of the revised manuscript, immediately following the References list.

**Authorship update**. During the revision process, we added Bing Pang (Graduate School of Asia-Pacific Studies, Waseda University, Tokyo 169-8050, Japan) as a co-author. Mr. Pang made substantive contributions to the revised manuscript by assisting with data processing, code organization, empirical analysis, robustness checks, and the preparation of reproducible supporting materials. All authors, including the newly added co-author, have reviewed and approved the revised manuscript and agree with the updated authorship and author order. We also updated the author information, affiliations, and author-contribution details in the submission system as required.

Below, please find our point-by-point responses to the reviewers' comments.

**Responses to Reviewer 1**

***1. Comment****: Breakpoint Justification: The selection of 2020 as a structural breakpoint is theoretically motivated (platform growth transition), but a formal structural break test (e.g., Bai–Perron or Chow test) would strengthen empirical justification.*

Response: We thank the reviewer for this highly constructive comment. We completely agree that a formal structural break test is necessary to strengthen our empirical justification. In the revised manuscript, we have now conducted a formal Chow-type known-break test specifying January 1, 2020, as the analytical breakpoint. The robust joint test strongly rejects the null hypothesis of parameter stability across the pre- and post-2020 periods (F(6, 2461) = 12.507, p < 0.001), providing strong empirical support for our phase-specific estimation strategy. These formal test results and interpretations have been added to the Results section. (Please see Section 4.2 and Table 6, Page 35-39).

***2. Comment****: Causal Interpretation: The manuscript appropriately avoids strong causal claims; however, given the observational time-series design, this limitation should be emphasized more clearly in the discussion.*

Response: We thank the reviewer for this important observation. We agree and have further toned down causal wording throughout the manuscript, especially in the Abstract, Introduction, Results, and Conclusion. The revised text now consistently frames the findings as associations, co-movements, or regime-sensitive relationships rather than direct causal effects. We also expanded the Discussion and Limitations sections to state explicitly that the observational design does not permit strong causal inference and to acknowledge possible reverse causality and omitted-variable bias. (Please see Section 5.4 Limitations, Page 46).

***3. Comment****: Measurement Validity of Affective Polarization: The use of media-level sentiment dispersion as a proxy for platform-mediated affective dynamics is theoretically defended. Still, additional sensitivity checks (e.g., alternative dispersion metrics) would enhance robustness.*

Response: Thank you for this valuable methodological suggestion. We completely agree with this change. To enhance robustness, we have conducted additional sensitivity checks across multiple specifications of the dispersion proxy (standardized, winsorized, spike-excluded, level, and winsorized-level versions), reported in Table 8 and visualized in S1 Appendix. These specifications collectively address concerns about scaling, extreme observations, and isolated spike days. We retain the standard deviation as the principal dispersion measure for the methodological reasons outlined in Section 3.1; rank-based alternatives such as Gini or IQR are noted as a useful direction for future extension. (Please see Section 4.4 and Table 8, Page 41-43, as well as Supporting Information S1 Appendix).

***4. Comment****: Magnitude Interpretation: While statistical significance is reported, effect sizes should be contextualized substantively to help readers understand real-world implications.*

Response: Thank you for this insightful comment. We completely agree with this suggestion. We have revised the discussion to substantively contextualize the effect sizes, explicitly explaining what the increased persistence and specific coefficients mean for real-world platform governance and community interactions. (Please see Section 5.1 and 5.2, Page 43-46).

**Responses to Reviewer 2**

***1. Comment****: The formal terminology of main headings has to change in order to comply with the following 6 main sections/titles: 1. Introduction, 2. Literature Review, 3. Methodology and Analysis, 4. Results, 5. Discussion, 6. Conclusions Implications and Future Works. All other-typed headings can change to aforementioned, or to be fixed as subsections underneath the aforementioned 6 main sections.*

Response: We thank the reviewer for this clear guidance. We agree and have restructured the manuscript so that it now follows the six required main sections. All former major sections have been reorganized accordingly, and the remaining headings have been renumbered as subsections under the new structure. (Please see the revised manuscript structure throughout).

***2. Comment****: The legends/captions of all Figures can be placed at the bottom of their graph areas, not on the top of them.*

Response: We thank the reviewer for this helpful formatting suggestion. We agree and have standardized the figure captions so that all figure titles and notes are now placed directly at the bottom of their respective graph areas. Specifically, this formatting update has been applied to Fig 1. Theoretical Framework of Bigtech’s Disciplinary Mechanisms in Section 2.3, and Fig 2. User Growth Trends of Major Social Platforms (2012-2024) in Section 4.2. (Please see Pages 23 and 36).

***3. Comment****: The narrative of the study contains many bold-typed, but non-numbered, subheadings. For this reason, all non-numbered subheadings have to be numbered in a sequence, jointly with the aforementioned 6 main headings/sections of the revised manuscript.*

Response: Thank you for your careful reading and suggestion. We completely agree with this change. All non-numbered subheadings have now been sequentially numbered to align with the 6 main sections. (Please see Section 2.1.1, 2.1.2, 2.2.1, etc., throughout the manuscript).

***4. Comment****: Regarding the digital platforms and their role of regulating them in order to better address “structural mechanisms of behavioral normalization and affective manipulation beyond content moderation alone”, there should be a broader and updated international theoretical coverage, since the half of citations are dating back more than a decade ago of publishing, whereas the topic of digital technologies in social context has attracted plentiful research interest in the last 5 years of literature production.*

Response: Thank you for recommending these highly relevant and updated studies. We completely agree with this suggestion. We have carefully read these excellent works and integrated them into our manuscript to broaden our theoretical coverage on digital transformation, macro/micro-economic impacts, uncertainty contexts, and environmental/social sustainability. (Please see Introduction, pp. 3-4; Section 2.1.1, pp. 12-13; Section 4.2, pp. 37-38; Section 6, pp. 52-53).

***5. Comment****: Almost all subsections 5.2-5.5 contain non cross-citing information, thus, checking and citing them in a more systematic manner can better validate the discussion on findings.*

Response: We thank the reviewer for this helpful comment. We agree and have revised the Results and Discussion so that the empirical findings are now interpreted in a more systematic manner, with clearer links to the theoretical framework and the existing literature. The discussion of each major result now connects the observed pattern to the relevant mechanism, rather than leaving the subsections isolated. (Please see Section 5, Pages 43-48).

***6. Comment****: In a separate and autonomous Discussion section authors are recommended to structure 2 subsections in which the a) per type-findings, b) per time-findings in decades’ intervals among the whole examined period, to be conveyed. In such a way a more comprehensive understanding of findings can be obtained when reading the study. Up to 2 extra and cross-cited text pages can be devoted in structuring this Discussion section.*

Response: We thank the reviewer for this constructive recommendation. We agree and have created a separate, autonomous Discussion section (Section 5) with clear subsections specifically dedicated to '5.1 Findings by Time Phase' and '5.2 Findings by Technology Type' to comprehensively convey our results. (Please see Section 5.1 and Section 5.2, Pages 43-46).

**Responses to Reviewer 3**

***1. Comment****: Causal language vs. correlational evidence. The abstract and conclusion repeatedly employ verbs such as “organize,” “reorganize,” “shape,” and “function as institutional actors that … through affective governance.” The VAR/Granger analysis demonstrates temporal precedence and regime-dependent associations but cannot establish that platform technologies cause polarization (or vice versa). Endogeneity is plausible: rising polarization may itself stimulate Big Tech R&D in recommendation and moderation technologies. I recommend toning down causal claims to “coincide with,” “are associated with,” or “co-evolve with,” and adding a dedicated paragraph in the Discussion on reverse causality, omitted-variable bias, and the limitations of publication proxies as measures of deployed platform mechanisms.*

Response: We thank the reviewer for this important and well-taken point. We agree and have toned down causal language throughout the manuscript, especially in the Abstract, Introduction, Results, Discussion, and Conclusion. The revised text now consistently frames the findings as associations and phase-sensitive relationships rather than direct causal effects. Additionally, we have added a dedicated paragraph in the Discussion section to explicitly address reverse causality, omitted variable bias, and proxy limitations. (Please see Section 5.4 Limitations, Page 46).

***2. Comment****: Validity of the affective-polarization proxy. Using the standard deviation of GDELT news-sentiment scores as a daily indicator of “affective polarization in the information environment” is clever and publicly replicable, yet it remains one step removed from platform users. GDELT aggregates global news tone; platforms operate in closed ecosystems with their own amplification logics. The manuscript justifies the proxy well but should more explicitly discuss (a) why news-tone dispersion is a valid upstream indicator of platform-mediated affective dynamics, (b) potential mismatches between journalistic sentiment and social-media user emotion, and (c) comparisons with alternative proxies (e.g., Twitter or Facebook emotion scores from prior studies). An edge-case consideration: GDELT’s global scope may dilute country-specific effects; a robustness check restricting to U.S.-centric events would strengthen claims.*

Response: We thank the reviewer for this helpful suggestion. We agree and have strengthened the explanation of the proxy in the Methods and Discussion. The revised manuscript now clarifies that GDELT-based sentiment dispersion is used as a media-level indicator of affective dynamics in the platform-mediated information environment, not as a direct measure of individual psychological states. Specifically, in revised Section 3.1, we now explicitly compare our GDELT-based proxy with prior platform-trace emotion data (Brady et al. 2017 [Ref 82]; Bail et al. 2018 [Ref 83]; Rathje et al. 2021 [Ref 84]) and clarify that GDELT operates upstream of platform-internal engagement traces, providing public availability and longitudinal continuity at the cost of distance from individual user states. We also discuss the conceptual gap between journalistic sentiment and user emotion, and we completely agree that a global aggregate may inadvertently dilute specific national dynamics, such as those in the U.S. context. However, due to the lack of access to the raw, event-level metadata required for precise localized filtering in our current database, we were unable to conduct a separate U.S.-centric robustness check. We have followed your suggestion by explicitly adding this as a methodological limitation in the Discussion section and highlighting the need for localized filtering in future research. (See Section 5.4, Page 46).

***3. Comment****: Choice and justification of the 2020 breakpoint. The breakpoint is described as “theory-informed,” yet the exact theoretical rationale (algorithmic shifts, COVID-19, regulatory pressure, or the Capitol insurrection) is not fully elaborated. Contemporaneous macro-shocks could confound results. Future readers will ask whether findings are robust to alternative breakpoints (e.g., 2018 Cambridge Analytica aftermath or 2022 Musk-Twitter acquisition). Sensitivity analyses and a clearer narrative link between 2020 events and specific platform disciplinary mechanisms (e.g., increased emotional-reaction buttons, recommendation of short-form video) would address this.*

Response: We thank the reviewer for this important observation. We completely agree and have substantially strengthened the theoretical and empirical justification for treating 2020 as an analytic breakpoint. In the revised manuscript, we now explicitly outline four converging structural shifts around 2020: (i) the COVID-19 pandemic's impact on online discourse, (ii) the rapid scaling of short-form video and emotion-reaction affordances (e.g., TikTok, Reels), (iii) the scrutiny following the 2020 U.S. election/Jan 6 events, and (iv) user-growth saturation. Furthermore, we implemented a formal Chow-type structural break test, which statistically confirms that the dynamic relationship shifted significantly post-2020. This ensures the breakpoint is both contextually grounded and empirically validated. (Please see Section 4.2 and Table 6, Page 35-39).

***4. Comment****: Technology-categorization methodology. The clustering procedure (K-means + ChatGPT active learning) is promising but requires expanded reporting: exact number of abstracts, feature vector construction (TF-IDF? embeddings?), number of clusters chosen and validation metrics (silhouette score, human inter-rater reliability on a hold-out set), and how the 10 final categories map onto “front-end” vs. “back-end” disciplinary functions. The manuscript mentions “Type3 recs” and “emotion-oriented interaction dynamics” but does not provide the full mapping table in the main text (only implied in Supporting Information). Readers need this transparency to evaluate construct validity.*

Response**:** We thank the reviewer for this detailed and constructive suggestion. We agree that the original manuscript did not provide sufficient transparency regarding the technology-categorization procedure. In the revised manuscript, we expanded Section 4.1 to explain more explicitly that the ten technology categories are interpreted as two broad dimensions on the basis of the clustering keywords in Table 3 and Rodríguez and Ortún’s front-back technology framework, rather than as a strict one-to-one functional mapping and we added S1 Text, which reports the Web of Science retrieval strategy, screening protocol, feature construction, clustering procedure, AI-assisted active-learning protocol, prompt template, validation notes, and the mapping of the ten technology categories to disciplinary functions.

Specifically, we now clarify that the technology-categorization dataset contains 49,775 Web of Science abstracts affiliated with Apple, Amazon, Facebook/Meta, Google/Alphabet, and Microsoft. Each abstract was treated as one text unit. The feature-vector construction used SentenceTransformer-based abstract embeddings, with UMAP dimensionality reduction before clustering. TF-IDF or class-based TF-IDF keywords were used for cluster interpretation rather than as the primary clustering feature. The initial technology categories were generated using MiniBatchKMeans, with the final number of clusters set to k=10. We also added further information on validation and refinement. The clustering procedure used WCSS / elbow-style diagnostics, silhouette-based uncertainty review, and interpretability checks. Low-confidence or boundary cases were then reviewed through a six-round active-learning process, with approximately 30 abstracts reviewed per round. ChatGPT was used as an auxiliary labeling and category-interpretation tool in later rounds, following a pre-specified labeling guideline and prompt template. Finally, we revised the category descriptions to avoid misleading labels. We also clarify that the front-end/back-end distinction is an interpretive heuristic rather than a rigid one-to-one functional classification. These revisions make the NLP-derived categories and their relationship to the platform-disciplinary framework more transparent, thereby strengthening construct validity. (Please see Section 4.1 and S1 text, Page 31)

5. ***Comment****: Omitted robustness and alternative specifications. Beyond the diagnostics noted in point 2, consider: (i) different sentiment-dispersion metrics (Gini, inter-quartile range), (ii) log-transformation or differencing of technology-intensity series, (iii) inclusion of news-volume controls, and (iv) subsample analyses excluding major news spikes. These would rule out artefactual results.*

Response: We thank the reviewer for this helpful suggestion. We agree that alternative dispersion measures can be useful for assessing proxy sensitivity. In revising the manuscript, we focused on robustness checks that are most directly aligned with the empirical object of this study: the temporal dynamics of daily media-level affective dispersion. Since the main research question concerns whether affective dispersion becomes more persistent after the 2020 transition period, we implemented sensitivity checks that directly test whether this finding depends on scaling choices, extreme first-difference observations, isolated spike days, or the use of first differences.

Specifically, we added standardized, winsorized, spike-excluded, level, and winsorized-level specifications of the affective-dispersion proxy. These checks are directly connected to the time-series structure of the model. The results show that the main phase-specific pattern is preserved across all first-difference-based specifications: the persistence coefficient increases from 0.149 to 0.664 in the baseline model, remains unchanged after standardization, increases from 0.350 to 0.662 after winsorization, and increases from 0.327 to 0.635 after excluding the top 1% of absolute daily changes. This indicates that the post-2020 increase in media-level affective persistence is not driven solely by the raw scale of the proxy, extreme observations, or isolated spike days.

We have revised the manuscript to clarify that the proxy captures media-level affective dispersion in the platform-mediated information environment, rather than individual-level psychological polarization. Accordingly, the added robustness checks evaluate the stability of the paper’s core time-series finding rather than reinterpreting the outcome as a different distributional inequality measure. (Please see Section 3.1 for the metric justification, and Section 4.4 and Table 8 for the comprehensive robustness checks, Pages 24-29 and 41-43)

Regarding (ii) log-transformation or differencing of the technology-intensity series: the seven-day moving average applied during construction (Section 3.1) already serves as the primary smoothing operation, and the ADF tests in Table 5 confirm stationarity of all type-level series at the 5% level (p < 0.05). Additional log-transformation is therefore not required for VAR estimability.

Regarding (iii) inclusion of news-volume controls: our dispersion-based proxy is constructed as a within-day statistic conditional on the set of events on that day. Once the within-day SD is computed, it is by construction normalized to event-set composition rather than absolute volume; days with fewer than 500 events are already excluded (Section 3.1). Adding daily volume as an external regressor would re-introduce a level confound that the within-day-SD design is intended to remove.

***6. Comment****: Minor Points. English polishing: break up some 50–60-word sentences; ensure consistent terminology (“affective polarization” vs. “emotional polarization” in title/abstract). Visuals: add a conceptual diagram of the platform-end-user community framework and a summary table of VAR results. Discussion: expand on generalizability (non-Western platforms, TikTok, WeChat), user heterogeneity, and ethical implications of using ChatGPT for labeling.*

Response: Thank you for these helpful detailed suggestions. We completely agree with all of them. We have thoroughly polished the English to break up long sentences and standardized the terminology to "emotional polarization" throughout. We have also ensured our conceptual diagram (Figure 1) is clearly presented and added a section in the Discussion to explore generalizability (including WeChat, TikTok) and the ethical implications of using ChatGPT. (Please see Section 5.3, Page 46).

Furthermore, we have added a dedicated "Future Works" paragraph in Section 6 that systematically extends these directions—covering generalizability to WeChat and TikTok, user-level heterogeneity, and methodological extensions—as natural follow-ups to our analytical design. (Please see Section 6, Page 52.)

Once again, we sincerely thank the editor and reviewers for their constructive feedback, which has substantially improved the manuscript.
