# Supplementary material for: Disciplining the digital public: Platform mechanisms and the dynamics of emotional polarization
Source: PLoS One. 2026 Aug 13;21(8):e0342143. doi: 10.1371/journal.pone.0342143 (PMC13472384; doi:10.1371/journal.pone.0342143)
Supplement: Supplementary file 4 [file pone.0342143.s007.docx]

**PLOS ONE**

**Reviewer Report**

*Disciplining the Digital Public: Platform Mechanisms and the Dynamics of Emotional Polarization*

**Manuscript Number:** PONE-D-26-02998R2 | Round: R2 (Revised)

**RECOMMENDATION: ACCEPT**

*The manuscript is now suitable for publication. All major and minor concerns from previous rounds have been comprehensively addressed with transparent, rigorous revisions.*

Prof Nirmal Kumar Ganguly and Dr Gautam Kumar Saha

AHERF , Delhi India

# 1. Adequacy of Response to Previous Review Comments

This is the second revision (R2). Reviewer 1 explicitly states that all major concerns from the prior round have been adequately addressed and recommends acceptance. Reviewer 3 provides a detailed, generous assessment recognizing substantial improvements in theoretical framing, empirical rigor, methodological transparency, and cautious interpretation, while offering five constructive minor suggestions. The authors have responded point-by-point and incorporated all five suggestions into the revised manuscript.

**Specific revisions implemented in R2:**

- **New consolidated summary Table 7** (early in Results) presenting phase-specific emotional-polarization persistence coefficients (pre- vs. post-2020) across baseline and five robustness specifications, plus selected cross-lag technology-category coefficients. This enables rapid appraisal of effect magnitudes without navigating multiple tables/appendices.
- **Revised Table 3** adding compact mapping of the ten technology categories to front-/back-end orientation and primary linkage to the three disciplinary mechanisms (behavioral standardization, cognitive dependency, emotionally structured differentiation). This improves immediate accessibility while full methodological details remain in S1 Text.
- **Publication-lag clarification** added in Section 3.1: technology-intensity series interpreted as publicly visible research-output signals of broader technological conditions (not direct measures of immediate feature deployment); post-2020 associations may partly reflect earlier R&D trajectories surfacing in the publication record.
- **Generalizability note** added (Sections 5.3 & 6): rapid scaling of emotion-reaction and infinite-scroll affordances on platforms such as TikTok (2018–2020) situated within the broader platform ecology; findings should be extended cautiously to non-Western platforms (e.g., WeChat, ByteDance/TikTok) with differing affordance configurations and governance logics.
- **Expanded policy implications paragraph** (Section 5.2): references the EU Digital Services Act’s systemic-risk assessment framework and explains how findings speak to structural mechanisms (algorithmic recommendation, interaction-feedback loops, affective engagement infrastructures) beyond content moderation, while maintaining cautious, observational language.

# 2. Technical Soundness and Support for Conclusions

**Yes – Technically sound observational study with conclusions appropriately supported by the data and analyses.** The core theoretical contribution—platform disciplinary mechanisms (interface design, algorithmic recommendation, interaction feedback) fostering behavioral standardization, cognitive dependency, and emotionally structured group differentiation—is timely and well-aligned with current debates on algorithmic amplification and affective governance. The platform-end user-community framework provides a coherent lens.

The empirical strategy is thoughtfully designed for an observational, proxy-based context: daily emotional-polarization indicator derived from GDELT news-sentiment dispersion (capturing information-environment polarization rather than individual psychological states), linked via time-series/VAR to technology-category intensity measures extracted via transparent NLP pipeline (SentenceTransformer embeddings, UMAP, MiniBatchKMeans k=10 validated by WCSS/silhouette, six-round active-learning + ChatGPT-assisted labeling with pre-specified guidelines) from ~50,000 Big Tech-affiliated Web of Science abstracts. The 2020 breakpoint is theory-informed (COVID discourse migration, short-form video scaling, post-election scrutiny, user-growth saturation) and rigorously validated by Chow test (F(6, 2461) = 12.507, p < 0.001).

**Key findings:** Emotional polarization becomes more persistent after 2020 and shows stronger associations with technological simplification and emotion-oriented interaction dynamics. These are reported with cautious associational language (“coincide with,” “are associated with,” “co-evolve with”) rather than causal claims, consistent with the observational design.

***Nuances & edge considerations:*** The authors openly discuss limitations (reverse causality, omitted-variable bias, distance between news-tone dispersion and individual psychological states, publication lag in WoS data). Robustness is extensively tested across standardized/winsorized/spike-excluded/level/winsorized-level versions and first-differenced specifications. The consolidated Table 7 and restructured Discussion (dedicated 5.1 Findings by Time Phase; 5.2 Findings by Technology Type with cross-referencing to theory) markedly improve interpretability and coherence.

# 3. Statistical Analysis Rigor

**Yes – Performed appropriately and rigorously.** The study employs time-series analysis with Vector Autoregression (VAR), structural-break testing (Chow test), and multiple robustness specifications. Pre- and post-2020 persistence coefficients and cross-lag associations are transparently reported. The new Table 7 provides a reader-friendly panel summary of key effect magnitudes across specifications and phases. Full coefficient estimates, standard errors, and supplementary figures remain available in S1 Appendix. No statistical concerns are apparent; the analytic approach matches the research questions and data structure.

# 4. Data Availability (PLOS Policy Compliance)

**Yes – Fully compliant; exemplary transparency.** The authors confirm that all data underlying the findings are fully available without restriction. S1 Data provides the derived daily time-series dataset; S1 Code supplies the scripts for data processing and statistical analyses; S1 Text documents the complete Web of Science retrieval, screening, clustering (Sentence Transformer + UMAP + Mini Batch K Means), active-learning protocol, and category-mapping procedures; S1 Appendix contains supplementary robustness results and figures. GDELT data are publicly available from the GDELT Project. Web of Science full abstract texts cannot be redistributed due to licensing, but the derived data, documentation, and code necessary to reproduce all reported analyses are provided. This level of open-science practice (especially the detailed NLP pipeline documentation) significantly strengthens construct validity and reproducibility claims.

# 5. Presentation, Clarity, and Standard English

**Yes – Clearly written in standard academic English.** Language is precise, professional, and accessible. The addition of the consolidated Table 7, revised Table 3 with mechanism mapping, and restructured Discussion subsections substantially improve readability and interpretive coherence. Minor presentational refinements requested by Reviewer 3 have been implemented. No typographical or grammatical issues requiring correction are noted in the provided materials. The manuscript meets PLOS ONE standards for clarity and presentation.

# 6. Detailed Review Comments to the Author

**Overall Assessment**

This revised manuscript represents a meaningful and timely contribution to the literature on platform governance, affective dynamics, and the structural mechanisms through which digital platforms shape public-opinion environments. The core theoretical innovation—platform disciplinary mechanisms (interface design, algorithmic recommendation, interaction feedback loops) fostering behavioral standardization, cognitive dependency, and emotionally structured group differentiation—is conceptually sharp and well-aligned with contemporary debates on algorithmic amplification, short-form video affordances, and the limits of content-moderation-centric regulation.

The empirical strategy, while necessarily proxy-based and observational, is thoughtfully designed and now further strengthened by formal structural-break testing (Chow test), extensive robustness checks across multiple specifications of the polarization proxy, and unusually transparent documentation of the NLP pipeline (S1 Text). The central finding—that emotional polarization in the information environment exhibits greater persistence after 2020 and stronger associations with technological simplification and emotion-oriented interaction dynamics—offers plausible correlational evidence consistent with the affective-governance thesis. The authors have consistently tempered causal language and expanded the Limitations section to address reverse causality, omitted-variable bias, proxy distance from individual psychology, and publication lag.

The R2 revisions directly and effectively address all five minor suggestions from Reviewer 3, resulting in a more accessible, interpretable, and policy-relevant manuscript. The addition of the consolidated VAR summary Table 7 is particularly helpful for readers. The manuscript is now ready for acceptance.

## Strengths of the Revised Manuscript (R2)

1. **Theoretical contribution & framework clarity:** The platform disciplinary mechanisms concept, linked to three concrete processes (standardization, dependency, differentiation), provides a coherent bridge between platform affordances/governance and affective polarization dynamics. Table 3 now makes this mapping immediately accessible in the main text.
2. **Methodological transparency & open science:** S1 Text’s detailed documentation of embeddings, dimensionality reduction, clustering validation, active-learning protocol (~30 abstracts/round × 6 rounds), and ChatGPT-assisted labeling with pre-specified guidelines is a model of reproducible qualitative-to-quantitative text analysis. This significantly bolsters construct validity for the ten technology categories.
3. **Empirical rigor & robustness:** Chow test provides statistical grounding for the 2020 breakpoint. Multiple proxy specifications (standardized, winsorized, spike-excluded, first-differenced) and the new consolidated Table 7 demonstrate stability of the post-2020 persistence increase. Publication-lag nuance is now explicitly acknowledged.
4. **Cautious, evidence-aligned interpretation:** Consistent shift to associational language, explicit discussion of endogeneity/proxy limitations, and careful framing of policy implications (observational evidence informing structural-mechanism attention under frameworks such as the EU DSA) align rhetorical claims with the observational design.
5. **Improved readability & coherence:** Dedicated subsections 5.1 (Findings by Time Phase) and 5.2 (Findings by Technology Type) with systematic cross-referencing to theory, plus the early Results summary Table 7, eliminate earlier impressions of isolated findings and enable quick appraisal of effect magnitudes.

## Remaining Minor / Optional Suggestions

No substantive issues remain. The following are very minor presentational or forward-looking suggestions for the authors’ optional consideration prior to final proofing:

- **Table/figure numbering & cross-references:** Confirm that all in-text references to the new Table 7 and revised Table 3 are correctly numbered and that supplementary tables in S1 Appendix retain clear, non-conflicting numbering.
- **Policy implications brevity:** The expanded paragraph referencing the EU DSA is appropriately cautious; if the journal imposes strict word limits, the authors could consider moving one supporting clause to a footnote while retaining the core linkage to systemic-risk assessment of structural mechanisms.
- **Future-work specificity:** The call for extension to WeChat, TikTok/ByteDance, etc., is well-placed. A single additional sentence sketching one concrete comparative dimension (e.g., differing infinite-scroll vs. algorithmic curation emphases) could further sharpen the agenda without lengthening the section materially.
- **Final proofread:** A light final pass to ensure uniform spacing, quotation formatting, and reference style (Vancouver/NLM) is recommended, though the manuscript already appears polished.

## Conclusion of Review

I commend the authors for a thorough, responsive, and high-quality revision. The manuscript now offers a conceptually innovative, empirically rigorous, and transparently documented contribution to understanding how platform disciplinary mechanisms may co-evolve with emotional polarization dynamics in the information environment. All reviewer and journal requirements have been met. I recommend acceptance without further substantive revision. The minor optional suggestions above are offered only to maximize clarity and policy resonance.

*This review was prepared independently. I have no conflicts of interest to declare.*

Review prepared for PLOS ONE editorial team – **PONE-D-26-02998R2** | All comments intended to support publication of high-quality, ethically sound research.
