# Supplementary material for: Disciplining the digital public: Platform mechanisms and the dynamics of emotional polarization
Source: PLoS One. 2026 Aug 13;21(8):e0342143. doi: 10.1371/journal.pone.0342143 (PMC13472384; doi:10.1371/journal.pone.0342143)
Supplement: Supplementary file 5 [file pone.0342143.s008.docx]

**Response to the Editor and Reviewers**

*Manuscript ID: PONE-D-26-02998*

Dear Dr. Cioffi and Reviewers,

Thank you very much for the opportunity to revise our manuscript, “Disciplining the Digital Public: Platform Mechanisms and the Dynamics of Emotional Polarization” (PONE-D-26-02998). We sincerely appreciate the editor’s and reviewers’ thoughtful comments and constructive feedback. We have carefully considered all remarks and revised the manuscript accordingly. We believe these revisions have improved the clarity, rigor, and overall presentation of the paper.

Below, we provide our point-by-point responses to the reviewers’ comments.

**Responses to Reviewer 1**

***1. Comment****: All comments have been addressed. The reviewer states that the major concerns raised in the previous round have been adequately addressed and recommends acceptance.*

**Response:**

We sincerely thank Reviewer 1 for this positive evaluation and for acknowledging the substantial improvements made in the revised manuscript. We are very pleased that the revisions have satisfactorily addressed the major concerns raised in the previous round, including the justification of the 2020 breakpoint, the more cautious use of causal language, the robustness checks for the emotional polarization proxy, and the expanded methodological description. We greatly appreciate the reviewer’s supportive assessment.

**Responses to Reviewer 3**

***Reviewer 3:*** *This revised manuscript represents a meaningful contribution to the growing literature on platform governance, affective dynamics, and the structural mechanisms through which digital platforms may shape public opinion environments. The core theoretical innovation—conceptualizing “platform disciplinary mechanisms” (interface design, algorithmic recommendation, and interaction feedback loops) as processes that foster behavioral standardization, cognitive dependency, and emotionally structured group differentiation—is timely and well-aligned with contemporary debates surrounding algorithmic amplification, short-form video affordances, and the limits of content-moderation-centric regulation.*

*The empirical strategy, while necessarily proxy-based and observational, is thoughtfully designed and now bolstered by formal structural-break testing, extensive robustness checks, and unusually transparent documentation of the NLP pipeline used to derive technology-intensity measures from nearly 50,000 Big Tech-affiliated abstracts. The central finding—that emotional polarization in the information environment (operationalized via GDELT news-sentiment dispersion) exhibits greater persistence after 2020 and stronger associations with technological simplification and emotion-oriented interaction dynamics—offers plausible correlational evidence consistent with the affective-governance thesis. The authors are to be commended for the care with which they have tempered causal language and for the expanded Limitations section that explicitly discusses reverse causality, omitted-variable bias, and the distance between news-tone dispersion and individual psychological states.*

*Strengths of the Revision*

*• Formal breakpoint validation: The Chow test result (F(6, 2461) = 12.507, p < 0.001) provides rigorous statistical grounding for treating 2020 as an analytic regime shift, complementing the rich contextual narrative around COVID-19 discourse migration, short-form video scaling (TikTok/Reels), post-election scrutiny, and user-growth saturation.*

*• Robustness of the polarization proxy: Testing standardized, winsorized, spike-excluded, level, and winsorized-level versions of the dispersion measure, and demonstrating stability of the post-2020 persistence increase across first-difference specifications, directly addresses earlier concerns about scaling artifacts and extreme observations.*

*• Methodological transparency (S1 Text): The new supporting text detailing SentenceTransformer embeddings, UMAP reduction, MiniBatchKMeans (k=10), WCSS/silhouette diagnostics, six-round active-learning protocol (~30 abstracts/round), ChatGPT-assisted labeling with pre-specified guidelines, and the front-end/back-end interpretive heuristic is a model of open science practice. This significantly strengthens construct validity claims for the ten technology categories.*

*• Restructured Discussion: The creation of dedicated subsections 5.1 (Findings by Time Phase) and 5.2 (Findings by Technology Type), together with systematic cross-referencing to the theoretical framework and prior literature, eliminates the earlier impression of isolated subsections and improves interpretive coherence.*

*• Cautious interpretation and limitations: The consistent reframing from causal verbs (“organize,” “shape,” “function as institutional actors”) to associational language (“coincide with,” “are associated with,” “co-evolve with”), plus the explicit paragraph on endogeneity and proxy limitations, aligns the rhetorical claims with the observational design.*

*Minor Suggestions for Further Improvement*

*While the revisions have addressed the major concerns, the following presentational and interpretive refinements would further strengthen the manuscript:*

**Response:**

We sincerely thank Reviewer 3 for the thoughtful and generous assessment of our revised manuscript, as well as for the helpful minor suggestions. We are very pleased that the revision was recognized as a meaningful contribution with improved theoretical framing, empirical rigor, and transparency. We also appreciate the reviewer’s detailed recognition of the improvements made in the breakpoint analysis, proxy robustness checks, methodological documentation, discussion structure, and caution in interpretation. In the revised manuscript, we have further refined the presentation according to the five minor suggestions, as detailed below.

***1. Comment****: Main-text technology category overview. Although S1 Text contains the full mapping, a compact summary table in the main text (perhaps as Table 3 or integrated into Section 4.1) listing the ten final categories, exemplar keywords/TF-IDF terms from each cluster, approximate front-end vs. back-end orientation, and linkage to the three disciplinary mechanisms (standardization, dependency, differentiation) would improve immediate accessibility for readers who do not consult the supplement. This need not duplicate every detail-two or three rows per dimension would suffice.*

**Response:**

We thank the reviewer for this helpful suggestion. We agree that the main text should provide a more immediately accessible overview of how the technology categories relate to the disciplinary-mechanism framework. In response, we revised Table 3 by adding a compact mapping between the ten technology categories, their front-/back-end orientation, and their primary linkage to the three disciplinary mechanisms: behavioral standardization, cognitive dependency, and emotionally structured differentiation. This revision allows readers to understand the interpretive bridge in the main text, while the fuller methodological details and validation notes remain available in S1 Text. (Please see Section 4.1 and Table 3, Pages 33~34)

***2. Comment****: Publication-lag and proxy interpretation. The use of WoS-indexed research outputs as a proxy for “technological conditions associated with platform governance” is reasonable but introduces a temporal lag (research often published 1-3 years after internal deployment decisions). A brief acknowledgment in Section 3.2 or 5.4 that the observed associations may partly reflect earlier (pre-2020) R&D trajectories surfacing in the publication record would add nuance, especially given the sharp post-2020 regime shift detected.*

**Response:**

We thank the reviewer for this valuable methodological observation. We agree that WoS-indexed research outputs should be interpreted carefully because they may involve a publication lag relative to internal R&D or deployment decisions. We have therefore added a clarification in Section 3.1 explaining that the technology-intensity series should be understood as publicly visible research-output signals of broader technological conditions, rather than as direct measures of immediate platform feature deployment. The revised text also notes that post-2020 associations may partly reflect R&D trajectories initiated earlier and subsequently appearing in the publication record. (Please see Section 3.1, Page 28)

***3. Comment****: Generalizability to non-Western platforms. The Discussion (5.3) and Future Works paragraph appropriately flag the need for extension to WeChat, TikTok (ByteDance), and other platforms whose affordances and recommendation logics may differ from the Apple-Amazon-Meta-Google-Microsoft ecosystem. A short additional sentence noting that TikTok’s rapid global scaling of emotion-reaction and infinite-scroll features after 2018-2020 may itself be a partial driver of the breakpoint (captured in the aggregate “emotion-oriented interaction dynamics” category) would help readers situate the Big-Five focus within a broader platform ecology.*

**Response:**

We thank the reviewer for this important suggestion. We have revised the discussion of generalizability to place the Big Five ecosystem within a broader global platform ecology. The revised text now notes that the rapid scaling of emotion-reaction affordances and infinite-scroll interfaces on platforms such as TikTok during and after 2018–2020 may form part of the broader platform ecology surrounding the observed post-2020 structural shift. We also clarify that the findings should be extended cautiously to other global and non-Western platforms, including WeChat and ByteDance/TikTok, where affordance configurations and governance logics may differ. (Please see Section 5.3 and Section 6, Pages 46~47 and 53)

***4. Comment****: VAR results summary table. A single, reader-friendly table (or small set of panels) early in the Results section that reports the key persistence coefficients and selected cross-lag associations for the baseline and preferred robustness specifications, pre- and post-2020, would allow quick appraisal of effect magnitudes without requiring readers to navigate between Table 6, Table 8, and the appendix figures.*

**Response:**

We thank the reviewer for this suggestion, which helps improve the readability of our results. To allow readers to appraise effect magnitudes quickly without having to navigate back and forth between multiple tables, we have added a consolidated summary table near the beginning of the Results section (new Table 7). This table combines the core phase-specific VAR results and proxy-sensitivity results in a panel format: Panel A reports the emotional-polarization persistence coefficient (Dstd, lag 1) under the baseline specification and five robustness specifications, with its pre- and post-2020 values and their difference; Panel B reports the lag-1 coefficients of the selected technology categories (Type0, Type3, Type4, Type5) on emotional polarization in the baseline VAR, together with their pre- and post-2020 changes. All values in the table are drawn directly from the existing VAR estimates and proxy-sensitivity checks; the table introduces no new analysis and serves only as a consolidated quick reference. The full coefficient estimates, standard errors, and supplementary robustness results remain available in S1 Appendix. We have also added a brief signpost at the beginning of Section 4.3 directing readers to this summary table for a rapid comparison of effect magnitudes across phases and specifications. (Please see Section 4.3 and the new Table 7, Pages 40~41)

5. ***Comment****: Policy implications paragraph. The Conclusion and the final paragraph of Section 5.2 already gesture toward regulatory attention to structural mechanisms beyond content moderation. Expanding this into one concise, evidence-linked sentence or short paragraph-e.g., referencing the EU Digital Services Act’s systemic-risk assessment obligations or analogous debates in other jurisdictions-would increase the manuscript’s relevance to ongoing policy conversations without overstating causal claims.*

**Response:**

We thank the reviewer for this constructive recommendation. We agree that the manuscript can more clearly connect the findings to ongoing policy debates without overstating causal claims. We have therefore expanded the policy implications paragraph by referring to the EU Digital Services Act’s systemic-risk assessment framework and by explaining how our findings speak to structural platform mechanisms beyond content moderation, including algorithmic recommendation, interaction-feedback loops, and affective engagement infrastructures. We have kept the interpretation cautious by emphasizing that the study provides observational and proxy-based evidence rather than direct causal proof. (Please see Section 5.2, Pages 45~46)

Once again, we sincerely thank the editor and reviewers for their constructive feedback. Their comments have substantially improved the manuscript.
