# Supplementary material for: Disciplining the digital public: Platform mechanisms and the dynamics of emotional polarization
Source: PLoS One. 2026 Aug 13;21(8):e0342143. doi: 10.1371/journal.pone.0342143 (PMC13472384; doi:10.1371/journal.pone.0342143)
Supplement: Peer review history

# Peer review: Decision Letter 0

**DOI:** 10.1371/journal.pone.0342143.r001

**Authors:** Andrea Cioffi

22 Mar 2026

Dear Dr. LU,

Thank you for submitting your manuscript to PLOS ONE. After careful consideration, we feel that it has merit but does not fully meet PLOS ONE’s publication criteria as it currently stands. Therefore, we invite you to submit a revised version of the manuscript that addresses the points raised during the review process.

We note that some of the comments of the reviewers refer to specific articles for you to cite. Please note that it is not mandatory that you cite these specific articles and you are welcome to seek alternatives manuscripts in the literature that are relevant to your manuscript’s content.

Please submit your revised manuscript by May 06 2026 11:59PM. If you will need more time than this to complete your revisions, please reply to this message or contact the journal office at plosone@plos.org. When you're ready to submit your revision, log on to https://www.editorialmanager.com/pone/ and select the 'Submissions Needing Revision' folder to locate your manuscript file.

- A letter that responds to each point raised by the academic editor and reviewer(s). You should upload this letter as a separate file labeled 'Response to Reviewers'.
- A marked-up copy of your manuscript that highlights changes made to the original version. You should upload this as a separate file labeled 'Revised Manuscript with Track Changes'.
- An unmarked version of your revised paper without tracked changes. You should upload this as a separate file labeled 'Manuscript'.

If you would like to make changes to your financial disclosure, please include your updated statement in your cover letter. Guidelines for resubmitting your figure files are available below the reviewer comments at the end of this letter.

If applicable, we recommend that you deposit your laboratory protocols in protocols.io to enhance the reproducibility of your results. Protocols.io assigns your protocol its own identifier (DOI) so that it can be cited independently in the future. For instructions see: https://journals.plos.org/plosone/s/submission-guidelines#loc-laboratory-protocols. Additionally, PLOS ONE offers an option for publishing peer-reviewed Lab Protocol articles, which describe protocols hosted on protocols.io. Read more information on sharing protocols at https://plos.org/protocols?utm_medium=editorial-email&utm_source=authorletters&utm_campaign=protocols.

We look forward to receiving your revised manuscript.

Kind regards,

Andrea Cioffi

Academic Editor

PLOS One

Journal Requirements:

When submitting your revision, we need you to address these additional requirements.

1. Please ensure that your manuscript meets PLOS ONE's style requirements, including those for file naming. The PLOS ONE style templates can be found at

https://journals.plos.org/plosone/s/file?id=wjVg/PLOSOne_formatting_sample_main_body.pdf and https://journals.plos.org/plosone/s/file?id=ba62/PLOSOne_formatting_sample_title_authors_affiliations.pdf

2. Please note that PLOS One has specific guidelines on code sharing for submissions in which author-generated code underpins the findings in the manuscript. In these cases, we expect all author-generated code to be made available without restrictions upon publication of the work. Please review our guidelines at https://journals.plos.org/plosone/s/materials-and-software-sharing#loc-sharing-code and ensure that your code is shared in a way that follows best practice and facilitates reproducibility and reuse.

3. Thank you for stating in your Funding Statement:

[The authors disclosed receipt of the following financial support for the research, authorship, and/or publication of this article: Grants-in-Aid for Scientific Research (KAKENHI C)Political Identify and Practices of Bigtech in the Digital Political Space (24K04704)].

Please provide an amended statement that declares *all* the funding or sources of support (whether external or internal to your organization) received during this study, as detailed online in our guide for authors at http://journals.plos.org/plosone/s/submit-now.  Please also include the statement “There was no additional external funding received for this study.” in your updated Funding Statement.

Please include your amended Funding Statement within your cover letter. We will change the online submission form on your behalf.

4. Please ensure that you include a title page within your main document. You should list all authors and all affiliations as per our author instructions and clearly indicate the corresponding author.

5. Please upload a copy of your Supporting Information files, to which you refer in your text on pages 26, 27, 42, and 43. If the figure is no longer to be included as part of the submission please remove all reference to it within the text. Please ensure that each Supporting Information file has a legend listed in the manuscript after the references list.

If the reviewer comments include a recommendation to cite specific previously published works, please review and evaluate these publications to determine whether they are relevant and should be cited. There is no requirement to cite these works unless the editor has indicated otherwise.

[Note: HTML markup is below. Please do not edit.]

Reviewers' comments:

Reviewer's Responses to Questions

**Comments to the Author**

1. Is the manuscript technically sound, and do the data support the conclusions?

Reviewer #1: Yes

Reviewer #2: Yes

Reviewer #3: Partly

**********

2. Has the statistical analysis been performed appropriately and rigorously?

Reviewer #1: Yes

Reviewer #2: Yes

Reviewer #3: Yes

**********

3. Have the authors made all data underlying the findings in their manuscript fully available?

The PLOS Data policy

Reviewer #1: Yes

Reviewer #2: Yes

Reviewer #3: Yes

**********

4. Is the manuscript presented in an intelligible fashion and written in standard English?

Reviewer #1: Yes

Reviewer #2: No

Reviewer #3: Yes

**********

Reviewer #1: I appreciate the opportunity to review this manuscript. The study tackles an important and timely question regarding how platform governance mechanisms intersect with affective polarization in digital public spheres. While technically sound, several aspects could benefit from further clarification:

Breakpoint Justification: The selection of 2020 as a structural breakpoint is theoretically motivated (platform growth transition), but a formal structural break test (e.g., Bai–Perron or Chow test) would strengthen empirical justification.

Causal Interpretation: The manuscript appropriately avoids strong causal claims; however, given the observational time-series design, this limitation should be emphasized more clearly in the discussion.

Measurement Validity of Affective Polarization: The use of media-level sentiment dispersion as a proxy for platform-mediated affective dynamics is theoretically defended. Still, additional sensitivity checks (e.g., alternative dispersion metrics) would enhance robustness.

Magnitude Interpretation: While statistical significance is reported, effect sizes should be contextualized substantively to help readers understand real-world implications.

Reviewer #2: 1.The formal terminology of main headings has to change in order to comply with the following 6 main sections/titles: 1.Introduction, 2.Literature Review, 3.Methodology and Analysis, 4.Results, 5.Discussion, 6.Conclusions Implications and Future Works. All other-typed headings can change to aforementioned, or to be fixed as subsections underneath the aforementioned 6 main sections.

2.The legends/captions of all Figures can be placed at the bottom of their graph areas, not on the top of them.

3.The narrative of the study contains many bold-typed, but non-numbered, subheadings. For this reason, all non-numbered subheadings have to be numbered in a sequence, jointly with the aforementioned 6 main headings/sections of the revised manuscript.

4.Regarding the digital platforms and their role of regulating them in order to better address “structural mechanisms of behavioral normalization and affective manipulation beyond content moderation alone”, there should be a broader and updated international theoretical coverage, since the half of citations are dating back more than a decade ago of publishing, whereas the topic of digital technologies in social context has attracted plentiful research interest in the last 5 years of literature production. Indicative studies (there is also a literature blossom of similar ones) that can be considered in the revised manuscript are the following: DOI: 10.1007/978-3-031-66801-2_2 , DOI: 10.3389/fenvs.2024.1371047 , DOI: 10.1007/978-3-031-30351-7_9 , DOI: 10.1007/978-3-031-30351-7_1 ,DOI: 10.3390/su13179577.

5.Almost all subsections 5.2-5.5 contain non cross-citing information, thus, checking and citing them in a more systematic manner can better validate the discussion on findings.

6.In a separate and autonomous Discussion section authors are recommended to structure 2 subsections in which the a) per type-findings, b) per time-findings in decades’ intervals among the whole examined period, to be conveyed. In such a way a more comprehensive understanding of findings can be obtained when reading the study. Up to 2 extra and cross-cited text pages can be devoted in structuring this Discussion section.

Reviewer #3: Strengths

This is a timely and ambitious contribution at the intersection of platform studies, computational social science, and affective-polarization research. The introduction of “platform disciplinary mechanisms” as a tripartite process (behavioral standardization via interface design, cognitive dependency via algorithmic recommendation, and emotionally structured group differentiation via interaction feedback) is genuinely novel and successfully bridges Foucaultian governmentality with contemporary digital-media theory. The empirical strategy—linking academic-publication intensity in Big Tech research domains to a media-level affective-polarization proxy—is creative and avoids the common reliance on proprietary platform trace data, thereby sidestepping many privacy and replicability barriers. The use of K-means clustering plus ChatGPT-assisted active learning for categorizing technology types (security, recommendation systems, emotion-oriented interaction, etc.) is methodologically innovative and transparently documented. Findings that affective polarization becomes more persistent post-2020 and more tightly coupled to front-end, emotion-oriented technology categories align with broader public and scholarly concerns about algorithmic amplification of outrage. The policy implications—advocating regulation that targets structural mechanisms rather than content moderation alone—are thoughtful and forward-looking.

Major Concerns (requiring revision)

Causal language vs. correlational evidence. The abstract and conclusion repeatedly employ verbs such as “organize,” “reorganize,” “shape,” and “function as institutional actors that … through affective governance.” The VAR/Granger analysis demonstrates temporal precedence and regime-dependent associations but cannot establish that platform technologies cause polarization (or vice versa). Endogeneity is plausible: rising polarization may itself stimulate Big Tech R&D in recommendation and moderation technologies. I recommend toning down causal claims to “coincide with,” “are associated with,” or “co-evolve with,” and adding a dedicated paragraph in the Discussion on reverse causality, omitted-variable bias, and the limitations of publication proxies as measures of deployed platform mechanisms.

Validity of the affective-polarization proxy. Using the standard deviation of GDELT news-sentiment scores as a daily indicator of “affective polarization in the information environment” is clever and publicly replicable, yet it remains one step removed from platform users. GDELT aggregates global news tone; platforms operate in closed ecosystems with their own amplification logics. The manuscript justifies the proxy well but should more explicitly discuss (a) why news-tone dispersion is a valid upstream indicator of platform-mediated affective dynamics, (b) potential mismatches between journalistic sentiment and social-media user emotion, and (c) comparisons with alternative proxies (e.g., Twitter or Facebook emotion scores from prior studies). An edge-case consideration: GDELT’s global scope may dilute country-specific effects; a robustness check restricting to U.S.-centric events would strengthen claims.

Choice and justification of the 2020 breakpoint. The breakpoint is described as “theory-informed,” yet the exact theoretical rationale (algorithmic shifts, COVID-19, regulatory pressure, or the Capitol insurrection) is not fully elaborated. Contemporaneous macro-shocks could confound results. Future readers will ask whether findings are robust to alternative breakpoints (e.g., 2018 Cambridge Analytica aftermath or 2022 Musk-Twitter acquisition). Sensitivity analyses and a clearer narrative link between 2020 events and specific platform disciplinary mechanisms (e.g., increased emotional-reaction buttons, recommendation of short-form video) would address this.

Technology-categorization methodology. The clustering procedure (K-means + ChatGPT active learning) is promising but requires expanded reporting: exact number of abstracts, feature vector construction (TF-IDF? embeddings?), number of clusters chosen and validation metrics (silhouette score, human inter-rater reliability on a hold-out set), and how the 10 final categories map onto “front-end” vs. “back-end” disciplinary functions. The manuscript mentions “Type3 recs” and “emotion-oriented interaction dynamics” but does not provide the full mapping table in the main text (only implied in Supporting Information). Readers need this transparency to evaluate construct validity.

Omitted robustness and alternative specifications. Beyond the diagnostics noted in point 2, consider: (i) different sentiment-dispersion metrics (Gini, inter-quartile range), (ii) log-transformation or differencing of technology-intensity series, (iii) inclusion of news-volume controls, and (iv) subsample analyses excluding major news spikes. These would rule out artefactual results.

Minor Points

English polishing: break up some 50–60-word sentences; ensure consistent terminology (“affective polarization” vs. “emotional polarization” in title/abstract).

Visuals: add a conceptual diagram of the platform–end-user–community framework and a summary table of VAR results.

Discussion: expand on generalizability (non-Western platforms, TikTok, WeChat), user heterogeneity, and ethical implications of using ChatGPT for labeling.

No issues of dual publication, research ethics (no human subjects), or competing interests apparent.

**********

what does this mean?). If published, this will include your full peer review and any attached files.

If you choose “no”, your identity will remain anonymous but your review may still be made public.

**Do you want your identity to be public for this peer review?**  For information about this choice, including consent withdrawal, please see our Privacy Policy

Reviewer #1: No

Reviewer #2: **Yes:**  Grigorios L. Kyriakopoulos

Reviewer #3: No

**********

[NOTE: If reviewer comments were submitted as an attachment file, they will be attached to this email and accessible via the submission site. Please log into your account, locate the manuscript record, and check for the action link "View Attachments". If this link does not appear, there are no attachment files.]

To ensure your figures meet our technical requirements, please review our figure guidelines: https://journals.plos.org/plosone/s/figures

You may also use PLOS’s free figure tool, NAAS, to help you prepare publication quality figures: https://journals.plos.org/plosone/s/figures#loc-tools-for-figure-preparation.

NAAS will assess whether your figures meet our technical requirements by comparing each figure against our figure specifications.

# Author comment: Author response to Decision Letter 1

**DOI:** 10.1371/journal.pone.0342143.r002

9 May 2026

Response to the Editor and Reviewers

Manuscript ID: PONE-D-26-02998

Dear Dr. Cioffi and Reviewers,

Thank you very much for the opportunity to revise our manuscript, "Disciplining the Digital Public: Platform Mechanisms and the Dynamics of Emotional Polarization" (PONE-D-26-02998). We would like to express our deepest gratitude to you and the three anonymous reviewers for the highly constructive criticisms and insightful comments. We have carefully studied all the comments and addressed them through targeted revisions, additional analyses, expanded methodological documentation, and clearer discussion of limitations. We believe that these revisions have substantially improved the clarity, rigor, and theoretical depth of our paper.

First of all, we have carefully reviewed all five Journal Requirements and revised the manuscript and supporting materials accordingly to comply with PLOS ONE's style, file-naming, code-sharing, and submission standards. In specific, regarding Requirement 3, the amended Funding Statement (declaring all sources of support and explicitly noting that no additional external funding was received) is included in our cover letter as requested. Regarding Requirement 5, a complete legend for each Supporting Information file (S1 Text, S1 Appendix, S1 Data, and S1 Code) has been added at the end of the revised manuscript, immediately following the References list.

Authorship update. During the revision process, we added Bing Pang (Graduate School of Asia-Pacific Studies, Waseda University, Tokyo 169-8050, Japan) as a co-author. Mr. Pang made substantive contributions to the revised manuscript by assisting with data processing, code organization, empirical analysis, robustness checks, and the preparation of reproducible supporting materials. All authors, including the newly added co-author, have reviewed and approved the revised manuscript and agree with the updated authorship and author order. We also updated the author information, affiliations, and author-contribution details in the submission system as required.

Below, please find our point-by-point responses to the reviewers' comments.

Responses to Reviewer 1

1. Comment: Breakpoint Justification: The selection of 2020 as a structural breakpoint is theoretically motivated (platform growth transition), but a formal structural break test (e.g., Bai–Perron or Chow test) would strengthen empirical justification.

Response: We thank the reviewer for this highly constructive comment. We completely agree that a formal structural break test is necessary to strengthen our empirical justification. In the revised manuscript, we have now conducted a formal Chow-type known-break test specifying January 1, 2020, as the analytical breakpoint. The robust joint test strongly rejects the null hypothesis of parameter stability across the pre- and post-2020 periods (F(6, 2461) = 12.507, p < 0.001), providing strong empirical support for our phase-specific estimation strategy. These formal test results and interpretations have been added to the Results section. (Please see Section 4.2 and Table 6, Page 35-39).

2. Comment: Causal Interpretation: The manuscript appropriately avoids strong causal claims; however, given the observational time-series design, this limitation should be emphasized more clearly in the discussion.

Response: We thank the reviewer for this important observation. We agree and have further toned down causal wording throughout the manuscript, especially in the Abstract, Introduction, Results, and Conclusion. The revised text now consistently frames the findings as associations, co-movements, or regime-sensitive relationships rather than direct causal effects. We also expanded the Discussion and Limitations sections to state explicitly that the observational design does not permit strong causal inference and to acknowledge possible reverse causality and omitted-variable bias. (Please see Section 5.4 Limitations, Page 46).

3. Comment: Measurement Validity of Affective Polarization: The use of media-level sentiment dispersion as a proxy for platform-mediated affective dynamics is theoretically defended. Still, additional sensitivity checks (e.g., alternative dispersion metrics) would enhance robustness.

Response: Thank you for this valuable methodological suggestion. We completely agree with this change. To enhance robustness, we have conducted additional sensitivity checks across multiple specifications of the dispersion proxy (standardized, winsorized, spike-excluded, level, and winsorized-level versions), reported in Table 8 and visualized in S1 Appendix. These specifications collectively address concerns about scaling, extreme observations, and isolated spike days. We retain the standard deviation as the principal dispersion measure for the methodological reasons outlined in Section 3.1; rank-based alternatives such as Gini or IQR are noted as a useful direction for future extension. (Please see Section 4.4 and Table 8, Page 41-43, as well as Supporting Information S1 Appendix).

4. Comment: Magnitude Interpretation: While statistical significance is reported, effect sizes should be contextualized substantively to help readers understand real-world implications.

Response: Thank you for this insightful comment. We completely agree with this suggestion. We have revised the discussion to substantively contextualize the effect sizes, explicitly explaining what the increased persistence and specific coefficients mean for real-world platform governance and community interactions. (Please see Section 5.1 and 5.2, Page 43-46).

Responses to Reviewer 2

1. Comment: The formal terminology of main headings has to change in order to comply with the following 6 main sections/titles: 1. Introduction, 2. Literature Review, 3. Methodology and Analysis, 4. Results, 5. Discussion, 6. Conclusions Implications and Future Works. All other-typed headings can change to aforementioned, or to be fixed as subsections underneath the aforementioned 6 main sections.

Response: We thank the reviewer for this clear guidance. We agree and have restructured the manuscript so that it now follows the six required main sections. All former major sections have been reorganized accordingly, and the remaining headings have been renumbered as subsections under the new structure. (Please see the revised manuscript structure throughout).

2. Comment: The legends/captions of all Figures can be placed at the bottom of their graph areas, not on the top of them.

Response: We thank the reviewer for this helpful formatting suggestion. We agree and have standardized the figure captions so that all figure titles and notes are now placed directly at the bottom of their respective graph areas. Specifically, this formatting update has been applied to Fig 1. Theoretical Framework of Bigtech’s Disciplinary Mechanisms in Section 2.3, and Fig 2. User Growth Trends of Major Social Platforms (2012-2024) in Section 4.2. (Please see Pages 23 and 36).

3. Comment: The narrative of the study contains many bold-typed, but non-numbered, subheadings. For this reason, all non-numbered subheadings have to be numbered in a sequence, jointly with the aforementioned 6 main headings/sections of the revised manuscript.

Response: Thank you for your careful reading and suggestion. We completely agree with this change. All non-numbered subheadings have now been sequentially numbered to align with the 6 main sections. (Please see Section 2.1.1, 2.1.2, 2.2.1, etc., throughout the manuscript).

4. Comment: Regarding the digital platforms and their role of regulating them in order to better address “structural mechanisms of behavioral normalization and affective manipulation beyond content moderation alone”, there should be a broader and updated international theoretical coverage, since the half of citations are dating back more than a decade ago of publishing, whereas the topic of digital technologies in social context has attracted plentiful research interest in the last 5 years of literature production.

Response: Thank you for recommending these highly relevant and updated studies. We completely agree with this suggestion. We have carefully read these excellent works and integrated them into our manuscript to broaden our theoretical coverage on digital transformation, macro/micro-economic impacts, uncertainty contexts, and environmental/social sustainability. (Please see Introduction, pp. 3-4; Section 2.1.1, pp. 12-13; Section 4.2, pp. 37-38; Section 6, pp. 52-53).

5. Comment: Almost all subsections 5.2-5.5 contain non cross-citing information, thus, checking and citing them in a more systematic manner can better validate the discussion on findings.

Response: We thank the reviewer for this helpful comment. We agree and have revised the Results and Discussion so that the empirical findings are now interpreted in a more systematic manner, with clearer links to the theoretical framework and the existing literature. The discussion of each major result now connects the observed pattern to the relevant mechanism, rather than leaving the subsections isolated. (Please see Section 5, Pages 43-48).

6. Comment: In a separate and autonomous Discussion section authors are recommended to structure 2 subsections in which the a) per type-findings, b) per time-findings in decades’ intervals among the whole examined period, to be conveyed. In such a way a more comprehensive understanding of findings can be obtained when reading the study. Up to 2 extra and cross-cited text pages can be devoted in structuring this Discussion section.

Response: We thank the reviewer for this constructive recommendation. We agree and have created a separate, autonomous Discussion section (Section 5) with clear subsections specifically dedicated to '5.1 Findings by Time Phase' and '5.2 Findings by Technology Type' to comprehensively convey our results. (Please see Section 5.1 and Section 5.2, Pages 43-46).

Responses to Reviewer 3

1. Comment: Causal language vs. correlational evidence. The abstract and conclusion repeatedly employ verbs such as “organize,” “reorganize,” “shape,” and “function as institutional actors that … through affective governance.” The VAR/Granger analysis demonstrates temporal precedence and regime-dependent associations but cannot establish that platform technologies cause polarization (or vice versa). Endogeneity is plausible: rising polarization may itself stimulate Big Tech R&D in recommendation and moderation technologies. I recommend toning down causal claims to “coincide with,” “are associated with,” or “co-evolve with,” and adding a dedicated paragraph in the Discussion on reverse causality, omitted-variable bias, and the limitations of publication proxies as measures of deployed platform mechanisms.

Response: We thank the reviewer for this important and well-taken point. We agree and have toned down causal language throughout the manuscript, especially in the Abstract, Introduction, Results, Discussion, and Conclusion. The revised text now consistently frames the findings as associations and phase-sensitive relationships rather than direct causal effects. Additionally, we have added a dedicated paragraph in the Discussion section to explicitly address reverse causality, omitted variable bias, and proxy limitations. (Please see Section 5.4 Limitations, Page 46).

2. Comment: Validity of the affective-polarization proxy. Using the standard deviation of GDELT news-sentiment scores as a daily indicator of “affective polarization in the information environment” is clever and publicly replicable, yet it remains one step removed from platform users. GDELT aggregates global news tone; platforms operate in closed ecosystems with their own amplification logics. The manuscript justifies the proxy well but should more explicitly discuss (a) why news-tone dispersion is a valid upstream indicator of platform-mediated affective dynamics, (b) potential mismatches between journalistic sentiment and social-media user emotion, and (c) comparisons with alternative proxies (e.g., Twitter or Facebook emotion scores from prior studies). An edge-case consideration: GDELT’s global scope may dilute country-specific effects; a robustness check restricting to U.S.-centric events would strengthen claims.

Response: We thank the reviewer for this helpful suggestion. We agree and have strengthened the explanation of the proxy in the Methods and Discussion. The revised manuscript now clarifies that GDELT-based sentiment dispersion is used as a media-level indicator of affective dynamics in the platform-mediated information environment, not as a direct measure of individual psychological states. Specifically, in revised Section 3.1, we now explicitly compare our GDELT-based proxy with prior platform-trace emotion data (Brady et al. 2017 [Ref 82]; Bail et al. 2018 [Ref 83]; Rathje et al. 2021 [Ref 84]) and clarify that GDELT operates upstream of platform-internal engagement traces, providing public availability and longitudinal continuity at the cost of distance from individual user states. We also discuss the conceptual gap between journalistic sentiment and user emotion, and we completely agree that a global aggregate may inadvertently dilute specific national dynamics, such as those in the U.S. context. However, due to the lack of access to the raw, event-level metadata required for precise localized filtering in our current database, we were unable to conduct a separate U.S.-centric robustness check. We have followed your suggestion by explicitly adding this as a methodological limitation in the Discussion section and highlighting the need for localized filtering in future research. (See Section 5.4, Page 46).

3. Comment: Choice and justification of the 2020 breakpoint. The breakpoint is described as “theory-informed,” yet the exact theoretical rationale (algorithmic shifts, COVID-19, regulatory pressure, or the Capitol insurrection) is not fully elaborated. Contemporaneous macro-shocks could confound results. Future readers will ask whether findings are robust to alternative breakpoints (e.g., 2018 Cambridge Analytica aftermath or 2022 Musk-Twitter acquisition). Sensitivity analyses and a clearer narrative link between 2020 events and specific platform disciplinary mechanisms (e.g., increased emotional-reaction buttons, recommendation of short-form video) would address this.

Response: We thank the reviewer for this important observation. We completely agree and have substantially strengthened the theoretical and empirical justification for treating 2020 as an analytic breakpoint. In the revised manuscript, we now explicitly outline four converging structural shifts around 2020: (i) the COVID-19 pandemic's impact on online discourse, (ii) the rapid scaling of short-form video and emotion-reaction affordances (e.g., TikTok, Reels), (iii) the scrutiny following the 2020 U.S. election/Jan 6 events, and (iv) user-growth saturation. Furthermore, we implemented a formal Chow-type structural break test, which statistically confirms that the dynamic relationship shifted significantly post-2020. This ensures the breakpoint is both contextually grounded and empirically validated. (Please see Section 4.2 and Table 6, Page 35-39).

4. Comment: Technology-categorization methodology. The clustering procedure (K-means + ChatGPT active learning) is promising but requires expanded reporting: exact number of abstracts, feature vector construction (TF-IDF? embeddings?), number of clusters chosen and validation metrics (silhouette score, human inter-rater reliability on a hold-out set), and how the 10 final categories map onto “front-end” vs. “back-end” disciplinary functions. The manuscript mentions “Type3 recs” and “emotion-oriented interaction dynamics” but does not provide the full mapping table in the main text (only implied in Supporting Information). Readers need this transparency to evaluate construct validity.

Response: We thank the reviewer for this detailed and constructive suggestion. We agree that the original manuscript did not provide sufficient transparency regarding the technology-categorization procedure. In the revised manuscript, we expanded Section 4.1 to explain more explicitly that the ten technology categories are interpreted as two broad dimensions on the basis of the clusteri

Attachment Submitted filename: Response to Reviewers.docx

# Peer review: Decision Letter 1

**DOI:** 10.1371/journal.pone.0342143.r003

**Authors:** Andrea Cioffi

8 Jun 2026

Dear Dr. LU,

Thank you for submitting your manuscript to PLOS ONE. After careful consideration, we feel that it has merit but does not fully meet PLOS ONE’s publication criteria as it currently stands. Therefore, we invite you to submit a revised version of the manuscript that addresses the points raised during the review process.

Please submit your revised manuscript by Jul 23 2026 11:59PM. If you will need more time than this to complete your revisions, please reply to this message or contact the journal office at plosone@plos.org. When you're ready to submit your revision, log on to https://www.editorialmanager.com/pone/ and select the 'Submissions Needing Revision' folder to locate your manuscript file.

- A letter that responds to each point raised by the academic editor and reviewer(s). You should upload this letter as a separate file labeled 'Response to Reviewers'.
- A marked-up copy of your manuscript that highlights changes made to the original version. You should upload this as a separate file labeled 'Revised Manuscript with Track Changes'.
- An unmarked version of your revised paper without tracked changes. You should upload this as a separate file labeled 'Manuscript'.

If you would like to make changes to your financial disclosure, please include your updated statement in your cover letter. Guidelines for resubmitting your figure files are available below the reviewer comments at the end of this letter.

If applicable, we recommend that you deposit your laboratory protocols in protocols.io to enhance the reproducibility of your results. Protocols.io assigns your protocol its own identifier (DOI) so that it can be cited independently in the future. For instructions see: https://journals.plos.org/plosone/s/submission-guidelines#loc-laboratory-protocols. Additionally, PLOS ONE offers an option for publishing peer-reviewed Lab Protocol articles, which describe protocols hosted on protocols.io. Read more information on sharing protocols at https://plos.org/protocols?utm_medium=editorial-email&utm_source=authorletters&utm_campaign=protocols.

As the corresponding author, your ORCID iD is verified in the submission system and will appear in the published article. PLOS supports the use of ORCID, and we encourage all coauthors to register for an ORCID iD and use it as well. Please encourage your coauthors to verify their ORCID iD within the submission system before final acceptance, as unverified ORCID iDs will not appear in the published article. *Only*  the individual author can complete the verification step; PLOS staff *cannot*  verify ORCID iDs on behalf of authors.

We look forward to receiving your revised manuscript.

Kind regards,

Andrea Cioffi

Academic Editor

PLOS One

Journal Requirements:

If the reviewer comments include a recommendation to cite specific previously published works, please review and evaluate these publications to determine whether they are relevant and should be cited. There is no requirement to cite these works unless the editor has indicated otherwise.

Please review your reference list to ensure that it is complete and correct. If you have cited papers that have been retracted, please include the rationale for doing so in the manuscript text, or remove these references and replace them with relevant current references. Any changes to the reference list should be mentioned in the rebuttal letter that accompanies your revised manuscript. If you need to cite a retracted article, indicate the article’s retracted status in the References list and also include a citation and full reference for the retraction notice.

[Note: HTML markup is below. Please do not edit.]

Reviewers' comments:

Reviewer's Responses to Questions

**Comments to the Author**

Reviewer #1: All comments have been addressed

Reviewer #2: All comments have been addressed

Reviewer #3: All comments have been addressed

**********

2. Is the manuscript technically sound, and do the data support the conclusions?

Reviewer #1: Yes

Reviewer #2: (No Response)

Reviewer #3: Yes

**********

3. Has the statistical analysis been performed appropriately and rigorously?

Reviewer #1: Yes

Reviewer #2: (No Response)

Reviewer #3: Yes

**********

4. Have the authors made all data underlying the findings in their manuscript fully available?

The PLOS Data policy

Reviewer #1: Yes

Reviewer #2: (No Response)

Reviewer #3: Yes

**********

5. Is the manuscript presented in an intelligible fashion and written in standard English?

Reviewer #1: Yes

Reviewer #2: (No Response)

Reviewer #3: Yes

**********

Reviewer #1: I have reviewed the revised manuscript and the authors’ point-by-point responses. In my opinion, the authors have adequately addressed the major concerns raised during the previous round of review.

The justification for the 2020 breakpoint has been strengthened through both theoretical explanation and a formal Chow-type structural break test. The causal language has been appropriately toned down, and the manuscript now presents the findings as associations rather than causal effects. The authors have also added useful robustness checks for the emotional polarization proxy, clarified the limitations of using GDELT-based media-level sentiment dispersion, and expanded the methodological description of the technology-categorization procedure.

The revised manuscript is more clearly structured, the discussion is better connected to the empirical findings, and the limitations are now stated more transparently. The supporting information, including data, code, methodological documentation, and robustness results, also improves the reproducibility and transparency of the study.

Overall, the manuscript now satisfies the major criteria for publication in PLOS ONE. The study presents original research, the analyses are described in sufficient detail, the conclusions are appropriately supported by the data, and the manuscript is intelligible and substantially improved. I therefore recommend that the manuscript be accepted for publication.

Reviewer #2: (No Response)

Reviewer #3: This revised manuscript represents a meaningful contribution to the growing literature on platform governance, affective dynamics, and the structural mechanisms through which digital platforms may shape public opinion environments. The core theoretical innovation—conceptualizing “platform disciplinary mechanisms” (interface design, algorithmic recommendation, and interaction feedback loops) as processes that foster behavioral standardization, cognitive dependency, and emotionally structured group differentiation—is timely and well-aligned with contemporary debates surrounding algorithmic amplification, short-form video affordances, and the limits of content-moderation-centric regulation.

The empirical strategy, while necessarily proxy-based and observational, is thoughtfully designed and now bolstered by formal structural-break testing, extensive robustness checks, and unusually transparent documentation of the NLP pipeline used to derive technology-intensity measures from nearly 50,000 Big Tech-affiliated abstracts. The central finding—that emotional polarization in the information environment (operationalized via GDELT news-sentiment dispersion) exhibits greater persistence after 2020 and stronger associations with technological simplification and emotion-oriented interaction dynamics—offers plausible correlational evidence consistent with the affective-governance thesis. The authors are to be commended for the care with which they have tempered causal language and for the expanded Limitations section that explicitly discusses reverse causality, omitted-variable bias, and the distance between news-tone dispersion and individual psychological states.

Strengths of the Revision

• Formal breakpoint validation: The Chow test result (F(6, 2461) = 12.507, p < 0.001) provides rigorous statistical grounding for treating 2020 as an analytic regime shift, complementing the rich contextual narrative around COVID-19 discourse migration, short-form video scaling (TikTok/Reels), post-election scrutiny, and user-growth saturation.

• Robustness of the polarization proxy: Testing standardized, winsorized, spike-excluded, level, and winsorized-level versions of the dispersion measure, and demonstrating stability of the post-2020 persistence increase across first-difference specifications, directly addresses earlier concerns about scaling artifacts and extreme observations.

• Methodological transparency (S1 Text): The new supporting text detailing SentenceTransformer embeddings, UMAP reduction, MiniBatchKMeans (k=10), WCSS/silhouette diagnostics, six-round active-learning protocol (~30 abstracts/round), ChatGPT-assisted labeling with pre-specified guidelines, and the front-end/back-end interpretive heuristic is a model of open science practice. This significantly strengthens construct validity claims for the ten technology categories.

• Restructured Discussion: The creation of dedicated subsections 5.1 (Findings by Time Phase) and 5.2 (Findings by Technology Type), together with systematic cross-referencing to the theoretical framework and prior literature, eliminates the earlier impression of isolated subsections and improves interpretive coherence.

• Cautious interpretation and limitations: The consistent reframing from causal verbs (“organize,” “shape,” “function as institutional actors”) to associational language (“coincide with,” “are associated with,” “co-evolve with”), plus the explicit paragraph on endogeneity and proxy limitations, aligns the rhetorical claims with the observational design.

Minor Suggestions for Further Improvement

While the revisions have addressed the major concerns, the following presentational and interpretive refinements would further strengthen the manuscript:

1. Main-text technology category overview. Although S1 Text contains the full mapping, a compact summary table in the main text (perhaps as Table 3 or integrated into Section 4.1) listing the ten final categories, exemplar keywords/TF-IDF terms from each cluster, approximate front-end vs. back-end orientation, and linkage to the three disciplinary mechanisms (standardization, dependency, differentiation) would improve immediate accessibility for readers who do not consult the supplement. This need not duplicate every detail—two or three rows per dimension would suffice.

2. Publication-lag and proxy interpretation. The use of WoS-indexed research outputs as a proxy for “technological conditions associated with platform governance” is reasonable but introduces a temporal lag (research often published 1–3 years after internal deployment decisions). A brief acknowledgment in Section 3.2 or 5.4 that the observed associations may partly reflect earlier (pre-2020) R&D trajectories surfacing in the publication record would add nuance, especially given the sharp post-2020 regime shift detected.

3. Generalizability to non-Western platforms. The Discussion (5.3) and Future Works paragraph appropriately flag the need for extension to WeChat, TikTok (ByteDance), and other platforms whose affordances and recommendation logics may differ from the Apple–Amazon–Meta–Google–Microsoft ecosystem. A short additional sentence noting that TikTok’s rapid global scaling of emotion-reaction and infinite-scroll features after 2018–2020 may itself be a partial driver of the breakpoint (captured in the aggregate “emotion-oriented interaction dynamics” category) would help readers situate the Big-Five focus within a broader platform ecology.

4. VAR results summary table. A single, reader-friendly table (or small set of panels) early in the Results section that reports the key persistence coefficients and selected cross-lag associations for the baseline and preferred robustness specifications, pre- and post-2020, would allow quick appraisal of effect magnitudes without requiring readers to navigate between Table 6, Table 8, and the appendix figures.

5. Policy implications paragraph. The Conclusion and the final paragraph of Section 5.2 already gesture toward regulatory attention to structural mechanisms beyond content moderation. Expanding this into one concise, evidence-linked sentence or short paragraph—e.g., referencing the EU Digital Services Act’s systemic-risk assessment obligations or analogous debates in other jurisdictions—would increase the manuscript’s relevance to ongoing policy conversations without overstating causal claims.

**********

what does this mean?). If published, this will include your full peer review and any attached files.

If you choose “no”, your identity will remain anonymous but your review may still be made public.

**Do you want your identity to be public for this peer review?**  For information about this choice, including consent withdrawal, please see our Privacy Policy

Reviewer #1: No

Reviewer #2: **Yes:**  Grigorios L. Kyriakopoulos

Reviewer #3: No

**********

[NOTE: If reviewer comments were submitted as an attachment file, they will be attached to this email and accessible via the submission site. Please log into your account, locate the manuscript record, and check for the action link "View Attachments". If this link does not appear, there are no attachment files.]

To ensure your figures meet our technical requirements, please review our figure guidelines: https://journals.plos.org/plosone/s/figures

You may also use PLOS’s free figure tool, NAAS, to help you prepare publication quality figures: https://journals.plos.org/plosone/s/figures#loc-tools-for-figure-preparation.

NAAS will assess whether your figures meet our technical requirements by comparing each figure against our figure specifications.

# Author comment: Author response to Decision Letter 2

**DOI:** 10.1371/journal.pone.0342143.r004

21 Jun 2026

Response to the Editor and Reviewers

Manuscript ID: PONE-D-26-02998

Dear Dr. Cioffi and Reviewers,

Thank you very much for the opportunity to revise our manuscript, “Disciplining the Digital Public: Platform Mechanisms and the Dynamics of Emotional Polarization” (PONE-D-26-02998). We sincerely appreciate the editor’s and reviewers’ thoughtful comments and constructive feedback. We have carefully considered all remarks and revised the manuscript accordingly. We believe these revisions have improved the clarity, rigor, and overall presentation of the paper.

Below, we provide our point-by-point responses to the reviewers’ comments.

Responses to Reviewer 1

1. Comment: All comments have been addressed. The reviewer states that the major concerns raised in the previous round have been adequately addressed and recommends acceptance.

Response:

We sincerely thank Reviewer 1 for this positive evaluation and for acknowledging the substantial improvements made in the revised manuscript. We are very pleased that the revisions have satisfactorily addressed the major concerns raised in the previous round, including the justification of the 2020 breakpoint, the more cautious use of causal language, the robustness checks for the emotional polarization proxy, and the expanded methodological description. We greatly appreciate the reviewer’s supportive assessment.

Responses to Reviewer 3

Reviewer 3: This revised manuscript represents a meaningful contribution to the growing literature on platform governance, affective dynamics, and the structural mechanisms through which digital platforms may shape public opinion environments. The core theoretical innovation—conceptualizing “platform disciplinary mechanisms” (interface design, algorithmic recommendation, and interaction feedback loops) as processes that foster behavioral standardization, cognitive dependency, and emotionally structured group differentiation—is timely and well-aligned with contemporary debates surrounding algorithmic amplification, short-form video affordances, and the limits of content-moderation-centric regulation.

The empirical strategy, while necessarily proxy-based and observational, is thoughtfully designed and now bolstered by formal structural-break testing, extensive robustness checks, and unusually transparent documentation of the NLP pipeline used to derive technology-intensity measures from nearly 50,000 Big Tech-affiliated abstracts. The central finding—that emotional polarization in the information environment (operationalized via GDELT news-sentiment dispersion) exhibits greater persistence after 2020 and stronger associations with technological simplification and emotion-oriented interaction dynamics—offers plausible correlational evidence consistent with the affective-governance thesis. The authors are to be commended for the care with which they have tempered causal language and for the expanded Limitations section that explicitly discusses reverse causality, omitted-variable bias, and the distance between news-tone dispersion and individual psychological states.

Strengths of the Revision

• Formal breakpoint validation: The Chow test result (F(6, 2461) = 12.507, p < 0.001) provides rigorous statistical grounding for treating 2020 as an analytic regime shift, complementing the rich contextual narrative around COVID-19 discourse migration, short-form video scaling (TikTok/Reels), post-election scrutiny, and user-growth saturation.

• Robustness of the polarization proxy: Testing standardized, winsorized, spike-excluded, level, and winsorized-level versions of the dispersion measure, and demonstrating stability of the post-2020 persistence increase across first-difference specifications, directly addresses earlier concerns about scaling artifacts and extreme observations.

• Methodological transparency (S1 Text): The new supporting text detailing SentenceTransformer embeddings, UMAP reduction, MiniBatchKMeans (k=10), WCSS/silhouette diagnostics, six-round active-learning protocol (~30 abstracts/round), ChatGPT-assisted labeling with pre-specified guidelines, and the front-end/back-end interpretive heuristic is a model of open science practice. This significantly strengthens construct validity claims for the ten technology categories.

• Restructured Discussion: The creation of dedicated subsections 5.1 (Findings by Time Phase) and 5.2 (Findings by Technology Type), together with systematic cross-referencing to the theoretical framework and prior literature, eliminates the earlier impression of isolated subsections and improves interpretive coherence.

• Cautious interpretation and limitations: The consistent reframing from causal verbs (“organize,” “shape,” “function as institutional actors”) to associational language (“coincide with,” “are associated with,” “co-evolve with”), plus the explicit paragraph on endogeneity and proxy limitations, aligns the rhetorical claims with the observational design.

Minor Suggestions for Further Improvement

While the revisions have addressed the major concerns, the following presentational and interpretive refinements would further strengthen the manuscript:

Response:

We sincerely thank Reviewer 3 for the thoughtful and generous assessment of our revised manuscript, as well as for the helpful minor suggestions. We are very pleased that the revision was recognized as a meaningful contribution with improved theoretical framing, empirical rigor, and transparency. We also appreciate the reviewer’s detailed recognition of the improvements made in the breakpoint analysis, proxy robustness checks, methodological documentation, discussion structure, and caution in interpretation. In the revised manuscript, we have further refined the presentation according to the five minor suggestions, as detailed below.

1. Comment: Main-text technology category overview. Although S1 Text contains the full mapping, a compact summary table in the main text (perhaps as Table 3 or integrated into Section 4.1) listing the ten final categories, exemplar keywords/TF-IDF terms from each cluster, approximate front-end vs. back-end orientation, and linkage to the three disciplinary mechanisms (standardization, dependency, differentiation) would improve immediate accessibility for readers who do not consult the supplement. This need not duplicate every detail-two or three rows per dimension would suffice.

Response:

We thank the reviewer for this helpful suggestion. We agree that the main text should provide a more immediately accessible overview of how the technology categories relate to the disciplinary-mechanism framework. In response, we revised Table 3 by adding a compact mapping between the ten technology categories, their front-/back-end orientation, and their primary linkage to the three disciplinary mechanisms: behavioral standardization, cognitive dependency, and emotionally structured differentiation. This revision allows readers to understand the interpretive bridge in the main text, while the fuller methodological details and validation notes remain available in S1 Text. (Please see Section 4.1 and Table 3, Pages 33~34)

2. Comment: Publication-lag and proxy interpretation. The use of WoS-indexed research outputs as a proxy for “technological conditions associated with platform governance” is reasonable but introduces a temporal lag (research often published 1-3 years after internal deployment decisions). A brief acknowledgment in Section 3.2 or 5.4 that the observed associations may partly reflect earlier (pre-2020) R&D trajectories surfacing in the publication record would add nuance, especially given the sharp post-2020 regime shift detected.

Response:

We thank the reviewer for this valuable methodological observation. We agree that WoS-indexed research outputs should be interpreted carefully because they may involve a publication lag relative to internal R&D or deployment decisions. We have therefore added a clarification in Section 3.1 explaining that the technology-intensity series should be understood as publicly visible research-output signals of broader technological conditions, rather than as direct measures of immediate platform feature deployment. The revised text also notes that post-2020 associations may partly reflect R&D trajectories initiated earlier and subsequently appearing in the publication record. (Please see Section 3.1, Page 28)

3. Comment: Generalizability to non-Western platforms. The Discussion (5.3) and Future Works paragraph appropriately flag the need for extension to WeChat, TikTok (ByteDance), and other platforms whose affordances and recommendation logics may differ from the Apple-Amazon-Meta-Google-Microsoft ecosystem. A short additional sentence noting that TikTok’s rapid global scaling of emotion-reaction and infinite-scroll features after 2018-2020 may itself be a partial driver of the breakpoint (captured in the aggregate “emotion-oriented interaction dynamics” category) would help readers situate the Big-Five focus within a broader platform ecology.

Response:

We thank the reviewer for this important suggestion. We have revised the discussion of generalizability to place the Big Five ecosystem within a broader global platform ecology. The revised text now notes that the rapid scaling of emotion-reaction affordances and infinite-scroll interfaces on platforms such as TikTok during and after 2018–2020 may form part of the broader platform ecology surrounding the observed post-2020 structural shift. We also clarify that the findings should be extended cautiously to other global and non-Western platforms, including WeChat and ByteDance/TikTok, where affordance configurations and governance logics may differ. (Please see Section 5.3 and Section 6, Pages 46~47 and 53)

4. Comment: VAR results summary table. A single, reader-friendly table (or small set of panels) early in the Results section that reports the key persistence coefficients and selected cross-lag associations for the baseline and preferred robustness specifications, pre- and post-2020, would allow quick appraisal of effect magnitudes without requiring readers to navigate between Table 6, Table 8, and the appendix figures.

Response:

We thank the reviewer for this suggestion, which helps improve the readability of our results. To allow readers to appraise effect magnitudes quickly without having to navigate back and forth between multiple tables, we have added a consolidated summary table near the beginning of the Results section (new Table 7). This table combines the core phase-specific VAR results and proxy-sensitivity results in a panel format: Panel A reports the emotional-polarization persistence coefficient (Dstd, lag 1) under the baseline specification and five robustness specifications, with its pre- and post-2020 values and their difference; Panel B reports the lag-1 coefficients of the selected technology categories (Type0, Type3, Type4, Type5) on emotional polarization in the baseline VAR, together with their pre- and post-2020 changes. All values in the table are drawn directly from the existing VAR estimates and proxy-sensitivity checks; the table introduces no new analysis and serves only as a consolidated quick reference. The full coefficient estimates, standard errors, and supplementary robustness results remain available in S1 Appendix. We have also added a brief signpost at the beginning of Section 4.3 directing readers to this summary table for a rapid comparison of effect magnitudes across phases and specifications. (Please see Section 4.3 and the new Table 7, Pages 40~41)

5. Comment: Policy implications paragraph. The Conclusion and the final paragraph of Section 5.2 already gesture toward regulatory attention to structural mechanisms beyond content moderation. Expanding this into one concise, evidence-linked sentence or short paragraph-e.g., referencing the EU Digital Services Act’s systemic-risk assessment obligations or analogous debates in other jurisdictions-would increase the manuscript’s relevance to ongoing policy conversations without overstating causal claims.

Response:

We thank the reviewer for this constructive recommendation. We agree that the manuscript can more clearly connect the findings to ongoing policy debates without overstating causal claims. We have therefore expanded the policy implications paragraph by referring to the EU Digital Services Act’s systemic-risk assessment framework and by explaining how our findings speak to structural platform mechanisms beyond content moderation, including algorithmic recommendation, interaction-feedback loops, and affective engagement infrastructures. We have kept the interpretation cautious by emphasizing that the study provides observational and proxy-based evidence rather than direct causal proof. (Please see Section 5.2, Pages 45~46)

Once again, we sincerely thank the editor and reviewers for their constructive feedback. Their comments have substantially improved the manuscript.

Attachment Submitted filename: Response_to_Reviewers_auresp_2.docx

# Peer review: Decision Letter 2

**DOI:** 10.1371/journal.pone.0342143.r005

**Authors:** Andrea Cioffi

9 Jul 2026

<p>Disciplining the Digital Public Platform Mechanisms and the Dynamics of Emotional Polarization

PONE-D-26-02998R2

Dear Dr. LU,

We’re pleased to inform you that your manuscript has been judged scientifically suitable for publication and will be formally accepted for publication once it meets all outstanding technical requirements.

Within one week, you’ll receive an e-mail detailing the required amendments. When these have been addressed, you’ll receive a formal acceptance letter and your manuscript will be scheduled for publication.

An invoice will be generated when your article is formally accepted. Please note, if your institution has a publishing partnership with PLOS and your article meets the relevant criteria, all or part of your publication costs will be covered. Please make sure your user information is up-to-date by logging into Editorial Manager at Editorial Manager® and clicking the ‘Update My Information' link at the top of the page. For questions related to billing, please contact billing support.

If your institution or institutions have a press office, please notify them about your upcoming paper to help maximize its impact. If they’ll be preparing press materials, please inform our press team as soon as possible -- no later than 48 hours after receiving the formal acceptance. Your manuscript will remain under strict press embargo until 2 pm Eastern Time on the date of publication. For more information, please contact onepress@plos.org.

Kind regards,

Andrea Cioffi

Academic Editor

PLOS One

Additional Editor Comments (optional):

Reviewers' comments:

Reviewer's Responses to Questions

**Comments to the Author**

Reviewer #3: All comments have been addressed

**********

2. Is the manuscript technically sound, and do the data support the conclusions?

Reviewer #3: Yes

**********

3. Has the statistical analysis been performed appropriately and rigorously?

Reviewer #3: Yes

**********

4. Have the authors made all data underlying the findings in their manuscript fully available?

The PLOS Data policy

Reviewer #3: Yes

**********

5. Is the manuscript presented in an intelligible fashion and written in standard English?

Reviewer #3: Yes

**********

Reviewer #3: This revised manuscript represents a meaningful and timely contribution to the literature on platform governance, affective dynamics, and the structural mechanisms through which digital platforms shape public-opinion environments. The core theoretical innovation—platform disciplinary mechanisms (interface design, algorithmic recommendation, interaction feedback loops) fostering behavioral standardization, cognitive dependency, and emotionally structured group differentiation—is conceptually sharp and well-aligned with contemporary debates on algorithmic amplification, short-form video affordances, and the limits of content-moderation-centric regulation.

The empirical strategy, while necessarily proxy-based and observational, is thoughtfully designed and now further strengthened by formal structural-break testing (Chow test), extensive robustness checks across multiple specifications of the polarization proxy, and unusually transparent documentation of the NLP pipeline (S1 Text). The central finding—that emotional polarization in the information environment exhibits greater persistence after 2020 and stronger associations with technological simplification and emotion-oriented interaction dynamics—offers plausible correlational evidence consistent with the affective-governance thesis. The authors have consistently tempered causal language and expanded the Limitations section to address reverse causality, omitted-variable bias, proxy distance from individual psychology, and publication lag.

The R2 revisions directly and effectively address all five minor suggestions from Reviewer 3, resulting in a more accessible, interpretable, and policy-relevant manuscript. The addition of the consolidated VAR summary Table 7 is particularly helpful for readers. The manuscript is now ready for acceptance.

Strengths of the Revised Manuscript (R2)

1. Theoretical contribution & framework clarity: The platform disciplinary mechanisms concept, linked to three concrete processes (standardization, dependency, differentiation), provides a coherent bridge between platform affordances/governance and affective polarization dynamics. Table 3 now makes this mapping immediately accessible in the main text.

2. Methodological transparency & open science: S1 Text’s detailed documentation of embeddings, dimensionality reduction, clustering validation, active-learning protocol (~30 abstracts/round × 6 rounds), and ChatGPT-assisted labeling with pre-specified guidelines is a model of reproducible qualitative-to-quantitative text analysis. This significantly bolsters construct validity for the ten technology categories.

3. Empirical rigor & robustness: Chow test provides statistical grounding for the 2020 breakpoint. Multiple proxy specifications (standardized, winsorized, spike-excluded, first-differenced) and the new consolidated Table 7 demonstrate stability of the post-2020 persistence increase. Publication-lag nuance is now explicitly acknowledged.

4. Cautious, evidence-aligned interpretation: Consistent shift to associational language, explicit discussion of endogeneity/proxy limitations, and careful framing of policy implications (observational evidence informing structural-mechanism attention under frameworks such as the EU DSA) align rhetorical claims with the observational design.

5. Improved readability & coherence: Dedicated subsections 5.1 (Findings by Time Phase) and 5.2 (Findings by Technology Type) with systematic cross-referencing to theory, plus the early Results summary Table 7, eliminate earlier impressions of isolated findings and enable quick appraisal of effect magnitudes.

Remaining Minor / Optional Suggestions

No substantive issues remain. The following are very minor presentational or forward-looking suggestions for the authors’ optional consideration prior to final proofing:

• Table/figure numbering & cross-references: Confirm that all in-text references to the new Table 7 and revised Table 3 are correctly numbered and that supplementary tables in S1 Appendix retain clear, non-conflicting numbering.

• Policy implications brevity: The expanded paragraph referencing the EU DSA is appropriately cautious; if the journal imposes strict word limits, the authors could consider moving one supporting clause to a footnote while retaining the core linkage to systemic-risk assessment of structural mechanisms.

• Future-work specificity: The call for extension to WeChat, TikTok/ByteDance, etc., is well-placed. A single additional sentence sketching one concrete comparative dimension (e.g., differing infinite-scroll vs. algorithmic curation emphases) could further sharpen the agenda without lengthening the section materially.

• Final proofread: A light final pass to ensure uniform spacing, quotation formatting, and reference style (Vancouver/NLM) is recommended, though the manuscript already appears polished.

**********

what does this mean?). If published, this will include your full peer review and any attached files.

If you choose “no”, your identity will remain anonymous but your review may still be made public.

**Do you want your identity to be public for this peer review?**  For information about this choice, including consent withdrawal, please see our Privacy Policy

Reviewer #3: **Yes:**  Prof Nirmal Kumar Ganguly

**********

Attachment Submitted filename: PLOS_ONE_Reviewer Prof N K Ganguly_Report.docx

# Editor report: Acceptance letter

**DOI:** 10.1371/journal.pone.0342143.r006

**Authors:** Andrea Cioffi

PONE-D-26-02998R2

PLOS One

Dear Dr. LU,

I'm pleased to inform you that your manuscript has been deemed suitable for publication in PLOS One. Congratulations! Your manuscript is now being handed over to our production team.

At this stage, our production department will prepare your paper for publication. This includes ensuring the following:

* All references, tables, and figures are properly cited

* All relevant supporting information is included in the manuscript submission,

* There are no issues that prevent the paper from being properly typeset

You will receive further instructions from the production team, including instructions on how to review your proof when it is ready. Please keep in mind that we are working through a large volume of accepted articles, so please give us a few days to review your paper and let you know the next and final steps.

Lastly, if your institution or institutions have a press office, please let them know about your upcoming paper now to help maximize its impact. If they'll be preparing press materials, please inform our press team within the next 48 hours. Your manuscript will remain under strict press embargo until 2 pm Eastern Time on the date of publication. For more information, please contact onepress@plos.org.

You will receive an invoice from PLOS for your publication fee after your manuscript has reached the completed accept phase. If you receive an email requesting payment before acceptance or for any other service, this may be a phishing scheme. Learn how to identify phishing emails and protect your accounts at https://explore.plos.org/phishing.

If we can help with anything else, please email us at customercare@plos.org.

Thank you for submitting your work to PLOS ONE and supporting open access.

Kind regards,

PLOS ONE Editorial Office Staff

on behalf of

Dr. Andrea Cioffi

Academic Editor

PLOS One
